# Multi-omics identifies a VIM-associated microglial program linked to lactylation, ferroptosis, and neuroinflammation after spinal cord injury

**DOI:** 10.3389/fgene.2026.1902559

**Published:** 2026-08-27

**Authors:** Jiayu Chen, Kai Chen, Zucheng Huang, Jie Tong

**Affiliations:** 1 Department of Spinal Surgery, The First People’s Hospital of Chenzhou, Chenzhou, China; 2 Department of Orthopedics, The Second Affiliated Hospital and Yuying Children’s Hospital of Wenzhou Medical University, The Second School of Clinical Medicine, Wenzhou Medical University, Wenzhou, China; 3 Division of Spine Surgery, Department of Orthopaedics, Nanfang Hospital, Southern Medical University, Guangzhou, China

**Keywords:** ferroptosis, lactylation, microglia, spinal cord injury, vimentin

## Abstract

**Background:**

The secondary injury cascade following spinal cord injury (SCI) involves substantial metabolic reprogramming and immune responses. However, whether transcriptional signatures related to lactylation, ferroptosis, and neuroinflammation show coordinated changes after SCI, and which cellular states are associated with these signatures, remain unclear.

**Methods:**

Public bulk transcriptomic datasets (GSE47681 and GSE5296) and a single-cell RNA-seq dataset (GSE162610) were integrated. Bioinformatic analyses included single-sample gene set enrichment analysis (ssGSEA), weighted gene co-expression network analysis (WGCNA), protein-protein interaction analysis, pseudotime trajectory inference, and *in silico* perturbation analysis. A C5 unilateral contusion SCI mouse model was used for transcriptomic validation, behavioral assessments, Western blotting, and immunofluorescence. Human peripheral blood RNA-seq data (GSE151371) were used as an exploratory external reference for systemic SCI-associated expression patterns.

**Results:**

Bulk transcriptomic analysis revealed coordinated elevation of lactylation-related, ferroptosis-related, and inflammation-related signature scores after SCI, with the highest scores observed at 3 days post-injury. Single-cell RNA-seq localized these signatures predominantly to expanded disease-associated microglia (DAM)-like states. Network screening prioritized vimentin (Vim) as a candidate hub gene associated with the three signatures, and Vim-high pathological microglia exhibited an activated inflammatory-metabolic transcriptional state. In the SCI mouse model, VIM, pan-lysine lactylation (pan-Kla), acyl-CoA synthetase long-chain family member 4 (ACSL4), and interleukin-1β (IL-1β) were upregulated in whole-lesion spinal cord tissue. Immunofluorescence further showed increased VIM, pan-Kla, and ACSL4 signals with spatial overlap within IBA1-positive cells after SCI.

**Conclusion:**

Pathological DAM-like microglia represent major microglial states exhibiting concurrent lactylation-related, ferroptosis-related, and inflammatory signatures after SCI. VIM was prioritized as a candidate hub associated with this pathological microglial program. These findings provide a cellular and molecular framework for future mechanistic studies of secondary SCI.

## Graphical Abstract

**Figure:**
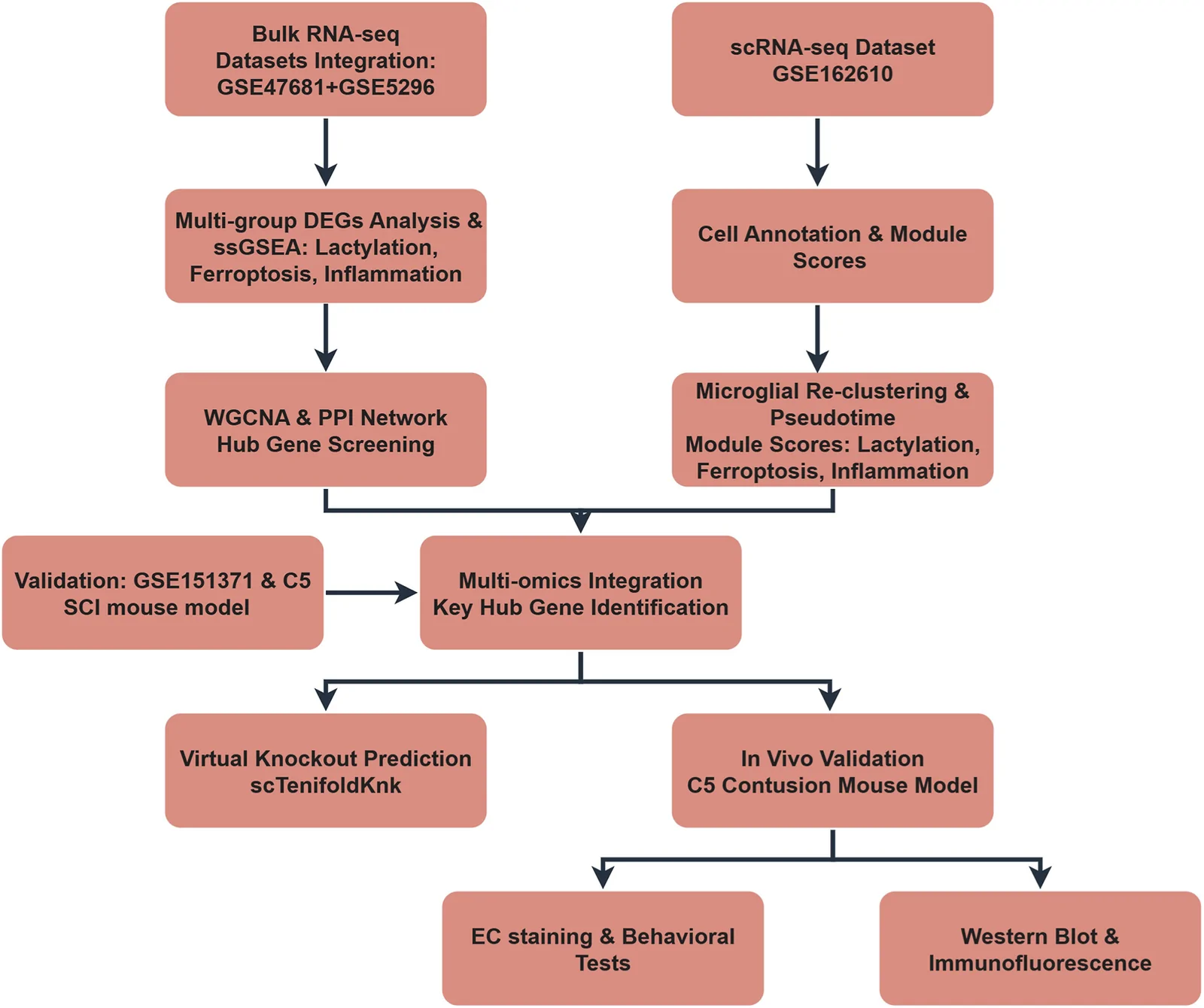

## 1 Introduction

Spinal cord injury (SCI) is a devastating insult to the central nervous system caused by high-energy mechanical forces, often resulting in permanent motor, sensory, and autonomic dysfunction below the injury level (Ahuja et al., 2017). With global population aging and the expansion of transportation networks, the incidence of SCI remains persistently high, imposing substantial burdens on patients, families, and healthcare systems worldwide (GBD, 2016 Traumatic Brain Injury and Spinal Cord Injury Collaborators, 2019). The pathophysiology of SCI is characterized by a distinct biphasic process: primary injury refers to the immediate mechanical disruption of spinal cord tissue and vasculature, while the secondary injury cascade persists for days to months and represents a critical, modifiable window for therapeutic intervention. Among the multifaceted events of secondary injury, excessive and persistent neuroinflammation—driven by the interplay of ischemia, hypoxia, oxidative stress, and immune cell infiltration—is widely recognized as the central pathological hub that propels progressive tissue necrosis and impedes neuroregeneration (David and Kroner, 2011).

As the resident immune cells of the central nervous system, microglia are the earliest responders to injury and the predominant regulators of neuroinflammation following SCI (Loane and Byrnes, 2010). Within minutes post-injury, microglia undergo profound morphological and transcriptional reprogramming, rapidly releasing pro-inflammatory cytokines such as tumor necrosis factor-alpha (TNF-α) and interleukin-1 beta (IL-1β) (Pineau and Lacroix, 2007). Subsequently, they establish complex intercellular communication networks with astrocytes and peripherally infiltrating macrophages, collectively shaping the immunological trajectory of the injury microenvironment (Pineau and Lacroix, 2007). Notably, the microglial response in SCI cannot be simply categorized as a binary “pro-inflammatory/anti-inflammatory” paradigm. Single-cell transcriptomic analyses have revealed remarkable heterogeneity and dynamic phenotypic transitions among microglia, with distinct subpopulations exhibiting functional specialization in phagocytosis, inflammatory output, and pro-repair potential (Keren-Shaul et al., 2017; Milich et al., 2021). Therefore, dissecting the pathological state transition of microglia and its upstream driving mechanisms at single-cell resolution is crucial for identifying novel therapeutic targets to intervene in secondary injury.

The immunological functions of microglia following SCI are inextricably linked to their profound metabolic reprogramming. Under the ischemic and hypoxic conditions of the injury microenvironment, microglia rapidly shift their energy metabolism from oxidative phosphorylation to aerobic glycolysis, a phenomenon known as the Warburg effect, to expeditiously meet the bioenergetic and biosynthetic demands of cellular activation (Lauro and Limatola, 2020). An inevitable consequence of this metabolic shift is the aberrant intracellular and extracellular accumulation of lactate, the terminal product of glycolysis. Historically, lactate has been regarded merely as a metabolic waste product. However, a seminal study by Zhang et al., in 2019 fundamentally altered this paradigm by demonstrating that lactate can serve as a substrate for covalent modification of histone lysine residues, giving rise to a novel epigenetic modification termed lysine lactylation (Kla), thereby directly coupling metabolic state to transcriptional regulation (Zhang et al., 2019). Lactylation is dynamically regulated by acyltransferases, such as p300/CREB-binding protein (CBP), and deacylases, including histone deacetylases 1–3 (HDAC1–3) and sirtuins 1–3 (SIRT1–3), and has been implicated in diverse biological processes, including immune cell fate determination and inflammatory gene expression (Liberti and Locasale, 2020).

The role of lactylation in SCI is increasingly being recognized. A 2024 single-cell RNA-sequencing study demonstrated that microglia undergo profound glycolytic remodeling post-injury, with lactylation-related genes such as Fabp5 and Lgals1 exhibiting dynamic expression changes during the subacute phase (Zhang et al., 2024). Galectin-1 (Lgals1) has also been reported to modulate microglial activation in neuroinflammatory settings (Starossom et al., 2012). Furthermore, a landmark study by Ge et al. published in Neuron in 2025 elegantly elucidated that injury-activated microglia/macrophages drive transcription of the chemokine Cxcl16 through histone lactylation, thereby recruiting CD8^+^ T lymphocytes and exacerbating neuronal loss and functional impairment. Notably, inhibition of glycolysis or blockade of histone lactylation partially reversed this pathogenic cascade (Ge et al., 2025). Collectively, these findings underscore the critical importance of lactylation in regulating neuroinflammation after SCI. However, a key question remains unanswered: beyond transcriptional activation of chemokines, does lactylation also modulate the inflammatory microenvironment by influencing the cell death modality of microglia?

Ferroptosis is a form of programmed cell death characterized by the accumulation of iron-dependent lipid peroxidation (Dixon et al., 2012). An increasing body of evidence has implicated ferroptosis as a critical mediator of secondary injury following SCI. Post-traumatic microvascular rupture leads to iron overload, while glutamate-mediated excitotoxicity concurrently inhibits the cystine/glutamate antiporter system xc^−^ antioxidant axis, thereby exposing neurons, oligodendrocytes, and even microglia to ferroptotic stress (Zhang et al., 2019). A recent study directly demonstrated that deletion of the Per2 gene in microglia inhibits ferroptosis through the peroxisome proliferator-activated receptor alpha (PPARα)-glutathione peroxidase 4 (Gpx4) axis, significantly improving motor functional recovery in SCI mice, thus providing direct evidence for the pathogenic role of microglial ferroptosis (Bie et al., 2025). Notably, ferroptosis is not merely a cell-autonomous death event. The lipid peroxidation products and damage-associated molecular patterns (DAMPs) released during ferroptotic death can further amplify neuroinflammation through pathways such as cyclic GMP–AMP synthase–stimulator of interferon genes (cGAS–STING), thereby establishing a ferroptosis-inflammation positive-feedback loop (Chen et al., 2023). Therefore, a critical and unresolved question arises: whether lactate-associated transcriptional programs after SCI are coordinated with ferroptosis-related and inflammatory signatures in microglia, thereby contributing to a pathological microglial state associated with secondary injury.

Based on the above research background, we proposed that after SCI, lactylation-related transcriptional programs may be coordinated with ferroptosis-associated molecular programs and inflammatory signaling in microglia. This coordinated transcriptional remodeling may contribute to pathological microglial state changes and secondary injury progression.

To test this hypothesis, we integrated public single-cell RNA-sequencing and bulk transcriptomic datasets, together with curated lactylation- and ferroptosis-related gene sets, and designed the following multi-omics analytical strategy: (1) At the bulk transcriptome level, datasets GSE47681 and GSE5296 were integrated to systematically evaluate the dynamic changes of lactylation-, ferroptosis-, and neuroinflammation-related transcriptional programs at different post-SCI time points. Differential expression analysis and functional enrichment analysis were performed to characterize the major molecular features of secondary injury. (2) Weighted gene co-expression network analysis (WGCNA) was employed to construct co-expression networks associated with SCI status and the three functional scores, through which key modules and candidate hub genes closely linked to the lactylation-ferroptosis-neuroinflammation axis were identified. Their expression stability was further assessed using transcriptomic data from an in-house SCI model, and their systemic SCI-associated expression patterns were explored using a human peripheral blood leukocyte dataset. (3) A single-cell RNA-sequencing dataset of SCI was utilized to construct a post-injury spinal cord cell atlas. The distribution patterns of the three functional scores and candidate hub genes across different cell types were analyzed to pinpoint the major pathologically relevant cell populations. (4) Microglia were subsequently extracted for sub-clustering, proportion dynamics analysis, functional scoring, and enrichment analysis to delineate the transcriptional signatures of pathological microglial subpopulations and their relationship with lactylation, ferroptosis, and inflammatory responses. (5) Pseudotime trajectory analysis was conducted to infer the transcriptional continuum of microglial states from homeostatic-like to pathologically activated states, and to observe the dynamic changes of key genes and the three functional scores along the pseudotime trajectory. (6) Within pathological microglia, differential expression analysis, correlation analysis, hub gene intersection and expression stratification were combined to identify a candidate molecule associated with lactylation-related, ferroptosis-related, and inflammatory transcriptional signatures, and to explore its associated functional network. (7) Finally, Western blot and immunofluorescence experiments were performed in a mouse SCI model to validate tissue-level protein changes and assess the spatial overlap of candidate signals with IBA1-positive cells. This study is expected to provide novel candidate molecules and a theoretical basis for microglia-targeted intervention and the treatment of secondary injury after SCI.

## 2 Materials and methods

### 2.1 Study design and data sources

This study integrated public bulk microarray transcriptomic datasets, a public single-cell RNA-sequencing dataset, and an in-house mouse SCI model to systematically investigate coordinated transcriptional signatures related to lactylation, ferroptosis, and neuroinflammation following SCI, and to identify the key cell subpopulations and hub genes involved. Public datasets were primarily obtained from the Gene Expression Omnibus (GEO). The bulk transcriptomic datasets included GSE47681, a microarray expression profiling dataset of mouse spinal cord covering sham, 1, 3, and 7 days post-injury (dpi) time points (Wu et al., 2013), and GSE5296, a time-series dataset of mouse SCI from which only the site of trauma samples were included. Specifically, naïve samples at 0 h, and moderately injured samples at 24 h, 72 h, and 7 days were extracted and defined as Sham, 1 dpi, 3 dpi, and 7 dpi, respectively (Faden and Hoffman, 2006). The single-cell RNA-sequencing dataset GSE162610 comprised 10 samples across four time points: uninjured, 1 dpi, 3 dpi, and 7 dpi (three biological replicates for uninjured, three for 1 dpi, two for 3 dpi, and two for 7 dpi) (Milich et al., 2021). To provide an exploratory external assessment of systemic SCI-associated expression patterns in humans, the peripheral blood leukocyte RNA-seq dataset GSE151371, which includes samples from healthy controls, non-central nervous system trauma controls, and patients with acute SCI, was incorporated (Kyritsis et al., 2021). In the present study, the comparison focused on healthy controls and patients with acute SCI. This dataset was used as an orthogonal peripheral immune reference and was not intended to directly represent VIM expression in central nervous system-resident microglia (Kyritsis et al., 2021). An in-house mouse C5 unilateral contusion SCI model was established for independent transcriptomic validation, behavioral assessments, Western blot, and immunofluorescence analyses. All animal experiments were performed in compliance with the ARRIVE guidelines and were approved by the Institutional Animal Care and Use Committee of Nanfang Hospital (Approval No. NFYY-2017-114).

### 2.2 Bulk transcriptomic data preprocessing and differential expression analysis

The expression matrices of GSE47681 and GSE5296 were imported into the R environment (version 4.2.1) for preprocessing. The transformation status of each source matrix was inspected before downstream analysis. The GSE47681 expression matrix was confirmed to be already log2-transformed; therefore, no additional log2 transformation was applied to this dataset. In contrast, the GSE5296 expression matrix was not log2-transformed based on its value distribution and was transformed using log2 (x + 1) before merging. For multiple probes mapping to the same gene, the average expression value was calculated. After harmonizing gene symbols and extracting common genes, the two matrices were merged. Batch effects were corrected using the ComBat function from the sva package, with dataset source defined as the batch and group information incorporated into the model matrix to preserve biological variation (Leek et al., 2012).

### 2.3 Functional gene set construction and enrichment analysis

Lactylation-, ferroptosis-, and neuroinflammation-related gene sets were manually curated from the Molecular Signatures Database (MSigDB) and previously published studies (Liberzon et al., 2011). Gene symbols were standardized according to official mouse gene nomenclature, and duplicated genes were removed before downstream analyses. The complete gene lists and their sources are provided in Supplementary Table S1. The DEGs at each time point were intersected with these thematic gene sets, and the overlapping patterns were visualized using UpSet plots. To evaluate overall pathway activity, single-sample gene set enrichment analysis (ssGSEA) was performed on the batch-corrected bulk expression matrix using the Gene Set Variation Analysis (GSVA) package, generating lactylation-related, ferroptosis-related, and inflammation-related signature scores for each sample (Hänzelmann et al., 2013). Spearman correlation analysis was conducted to assess associations among the three signature scores within SCI samples. Subsequently, DEGs at key time points were extracted, and Gene Ontology (GO) and Kyoto Encyclopedia of Genes and Genomes (KEGG) pathway enrichment analyses were performed using the clusterProfiler package (BH-adjusted P < 0.05) (Yu et al., 2012). In addition, gene set enrichment analysis (GSEA) was employed to evaluate the trend of pathway activity alterations at the transcriptome-wide level, with the significance threshold set at |normalized enrichment score (NES)| > 1 and adjusted P < 0.05 (Subramanian et al., 2005).

### 2.4 Weighted gene co-expression network analysis (WGCNA) and hub gene identification

The top 5,000 genes with the largest expression variance in the bulk matrix were selected to construct a scale-free weighted gene co-expression network using the WGCNA package (Langfelder and Horvath, 2008). SCI status and the three ssGSEA scores were used as phenotypic traits. The optimal soft-thresholding power was determined by the pickSoftThreshold function, and a signed network was constructed to calculate module eigengenes. Genes from the key modules significantly correlated with SCI status and the three functional scores were extracted, and scatter plots of module membership versus gene significance were generated. These key module genes were then intersected with the three previously constructed thematic gene sets, retaining candidate genes present in the key modules and overlapping with at least two functional gene sets. Subsequently, a protein–protein interaction (PPI) network was constructed for the overlapping candidate genes using the STRING database and Cytoscape software, and hub genes were identified based on node degree calculated by the cytoHubba plugin (Szklarczyk et al., 2023; Shannon et al., 2003; Chin et al., 2014).

### 2.5 Single-cell transcriptomic data preprocessing and cell type annotation

Raw sequencing data of GSE162610 were downloaded and analyzed using the Seurat package (version 5.3.0) (Milich et al., 2021; Hao et al., 2021). Quality control criteria were set as follows: genes expressed per cell > 300, genes detected in at least 5 cells, and mitochondrial gene percentage < 20%. Data were normalized and scaled via SCTransform, and the top 3,000 highly variable genes (HVGs) were selected, followed by principal component analysis (PCA) on the first 50 principal components (PCs). Batch effects across samples were corrected using the harmony package (Korsunsky et al., 2019). Cell clustering (resolution = 0.5, k = 20) and Uniform Manifold Approximation and Projection (UMAP) visualization were performed based on the Harmony-corrected space. Based on canonical marker genes, cells were classified into 13 major cell types: Microglia, Macrophage, Monocyte, dividing myeloid cells (Div-Myeloid), Neutrophil, Astrocyte, Oligodendrocyte, oligodendrocyte precursor cells (OPCs), Ependymal, Endothelial, Pericyte, Fibroblast, and Neuron (Milich et al., 2021). Cell composition proportions at each time point were calculated and displayed as stacked bar plots, and marker gene expression was visualized by DotPlot. The AddModuleScore function was used to compute lactylation, ferroptosis, and inflammation module scores at the single-cell level. The spatiotemporal expression patterns of the optimized core targets were visualized using FeaturePlot and boxplots to identify the major cell populations associated with the pathological signatures. SCTransform and Harmony were used for normalization, dimensionality reduction, integration, clustering, and visualization of the single-cell dataset. Harmony correction was applied to reduce unwanted variation during integration and UMAP visualization. For downstream interpretation, cell-level visualization was combined with sample-aware statistical summaries where appropriate, as described in the Statistical Analysis section.

### 2.6 Microglial subclustering and definition of pathological disease-associated microglia-like states

Microglia were extracted and subjected to a second round of normalization using SCTransform. The top 3,000 HVGs were selected, PCA was performed, and Harmony was applied for batch correction (dims = 1:11) (Korsunsky et al., 2019). Subclustering was conducted using FindNeighbors (k.param = 25), FindClusters (resolution = 0.1), and UMAP (n.neighbors = 25, min. dist = 0.1). Based on marker gene expression and functional characteristics, seven subpopulations were ultimately annotated: True Homeostatic microglia (MG) I, Activated Homeostatic-like MG I, Homeostatic MG II antigen-presenting cell-like (APC-like), Transitional MG, disease-associated microglia (DAM) lipid/metabolic, Inflammatory DAM, and interferon-responsive microglia (IFN-responsive MG) (Keren-Shaul et al., 2017; Masuda et al., 2019). The DAM lipid/metabolic and Inflammatory DAM subpopulations were merged and operationally defined as combined DAM, representing a study-defined pathological DAM-like microglial state, while True Homeostatic MG I served as the homeostatic reference. The proportional changes and functional scores across subpopulations were compared. Wilcoxon rank-sum tests implemented in Seurat were used for exploratory marker-gene screening between combined DAM and True Homeostatic MG I, primarily to support subcluster annotation and downstream functional enrichment. These cell-level marker analyses were interpreted as exploratory and were not used as standalone evidence for causal inference (min.pct = 0.10; screening criteria: adjusted P < 0.05 and |log2FC| ≥ 0.25). The resulting DEGs were subjected to GO, KEGG, and GSEA enrichment analyses (Yu et al., 2012; Subramanian et al., 2005).

### 2.7 Microglial subpopulation trajectory and pseudotime analysis

To infer the transcriptional state continuum of microglia after SCI, pseudotime trajectory analysis was performed on all microglia using the Monocle2 package, with dimensionality reduction via the DDRTree method (Qiu et al., 2017). True Homeostatic MG I cells from the sham group were set as the root node. The distribution of cells along the pseudotime trajectory was analyzed, and the dynamic changes of key genes and functional scores were evaluated. Spearman and Pearson correlations between lactylation, ferroptosis, and inflammation scores and pseudotime were calculated. Pseudotime-associated genes were identified using the differentialGeneTest function, and the top 50 genes were visualized in a heatmap to characterize transcriptional module remodeling along the inferred microglial state continuum.

### 2.8 Identification of the candidate VIM-associated hub gene and *in silico* perturbation analysis

Combined DAM cells from non-Sham groups were extracted and re-clustered to further characterize VIM-associated pathological microglial states. Re-clustering was performed using SCTransform, PCA, FindNeighbors, FindClusters, and UMAP, with dims = 1:15, resolution = 0.20, n. neighbors = 20, and min. dist = 0.20. To reduce the influence of single-cell dropout in Vim expression, combined DAM re-clusters were ranked according to cluster-level median Vim expression and operationally classified into Vim-low, Vim-mid, and Vim-high groups.

Significantly upregulated genes in combined DAM were first screened, and their Spearman correlations with the three single-cell-level signature scores were calculated. Candidate genes were further prioritized by intersection with hub genes derived from the bulk WGCNA-PPI analysis. This convergent screening strategy prioritized vimentin (Vim) as a candidate hub gene associated with lactylation-related, ferroptosis-related, and inflammation-related signature scores.

Vim-high and Vim-low clusters were compared using Wilcoxon rank-sum tests implemented in Seurat. These cell-level differential expression results were used as exploratory screening for functional annotation rather than standalone evidence of causal regulation. Vim-mid clusters were retained for visualization and module-score comparison but were not included in the Vim-high versus Vim-low DEG analysis. GO, KEGG, and GSEA enrichment analyses were then performed to characterize transcriptional programs associated with the Vim-high state.

To further explore putative VIM-associated network perturbations, the scTenifoldKnk algorithm was used for *in silico* Vim perturbation analysis (Osorio et al., 2022). The analysis was performed in Vim-high pathological microglia when sufficient cells were available; otherwise, all non-Sham combined DAM cells were used. Predicted perturbation-associated genes were intersected with Vim-high versus Vim-low DEGs, and PPI analysis was performed to nominate candidate VIM-associated network components. These *in silico* analyses were interpreted as hypothesis-generating and were not considered equivalent to experimental Vim loss-of-function validation.

### 2.9 Establishment of the C5 SCI animal model

Eight-week-old male C57BL/6J mice were randomly assigned to a Sham group (n = 20) or an SCI group (n = 20). Male mice were selected to maintain consistency with the established C5 unilateral contusion protocol used in our laboratory and the in-house validation cohorts. In this unilateral cervical hemicontusion model, spontaneous micturition and defecation were preserved; therefore, animal sex was not selected on the basis of bladder-management considerations. Mice were anesthetized using an anesthesia vaporizer with isoflurane, with induction at 3% in oxygen and maintenance at 1.5%–2.0% during surgery. After anesthesia was established, the C5 vertebral lamina was exposed. In the SCI group, the left hemi-lamina was removed, and a unilateral contusion was applied to the exposed dorsal surface of the spinal cord using a custom-built spinal cord impactor (impact velocity: 300 mm/s; depth: 1.2 mm; dwell time: 100 ms) (Huang et al., 2019). Injury parameters were recorded in real time by an integrated sensor and visualized to ensure procedural consistency. Mice in the Sham group underwent laminectomy only, without contusive injury. After wound closure, mice received 1 mL of sterile 0.9% saline by subcutaneous injection for fluid replacement. Enrofloxacin (5 mg/kg, subcutaneously) was administered once daily for 5 consecutive days for postoperative infection prophylaxis. Mice were allowed to recover in a temperature-controlled chamber maintained at approximately 25 °C until spontaneous mobility was restored, after which they were returned to their home cages. Animals were monitored daily for hydration status, food and water intake, wound condition, spontaneous activity, urinary and fecal output, and signs of infection or distress. Manual bladder expression was not required because spontaneous micturition and defecation were preserved. No postoperative mortality occurred before the predetermined experimental endpoints.

### 2.10 Anesthesia and euthanasia

All surgical procedures were performed under inhaled isoflurane anesthesia using an anesthesia vaporizer. Anesthesia was induced with 3% isoflurane in oxygen and maintained with 1.5%–2.0% isoflurane during surgery. Adequate anesthesia was confirmed by the absence of pedal withdrawal reflexes before surgical manipulation. For tissue collection, mice were deeply anesthetized with 5% isoflurane until loss of pedal withdrawal reflexes and respiratory arrest. For transcriptomic sequencing and Western blotting, mice were euthanized by cervical dislocation before spinal cord tissue harvesting. For immunofluorescence staining, deeply anesthetized mice were transcardially perfused with phosphate-buffered saline followed by 4% paraformaldehyde. Death was confirmed by cessation of respiration and heartbeat before tissue collection.

### 2.11 In-house SCI transcriptomic sequencing and external validation

To validate the findings from the bioinformatic screening, spinal cord tissues at the injury epicenter were collected from the SCI group at 3 dpi and 7 dpi, and from the Sham group (n = 3 per group). Total RNA was extracted using TRIzol reagent (Chomczynski and Sacchi, 1987). After quality assessment (RNA Integrity Number, RIN ≥ 7.0), library construction and sequencing were performed by Biomarker Technologies Co., Ltd. (Beijing, China) on the Illumina NovaSeq 6000 platform. After quality control, clean reads were aligned to the mouse reference genome (GRCm38/mm10) using HISAT2, and gene-level quantification was performed with featureCounts, followed by normalization using the FPKM (Fragments Per Kilobase of transcript per Million mapped reads) method (Kim et al., 2019; Liao et al., 2014; Mortazavi et al., 2008). Differential expression analysis was conducted using the limma-voom pipeline, with the group and batch information incorporated into the model. After batch effect removal, expression differences of core genes were visualized as Z-score normalized values (screening criteria: |log2FC| > 1 and adjusted P < 0.05), thereby providing an independent cohort validation of the public data findings (Ritchie et al., 2015). Furthermore, an exploratory comparison of candidate hub-gene expression between healthy controls and patients with acute SCI was performed using the GSE151371 peripheral blood leukocyte dataset (Kyritsis et al., 2021). This analysis was intended to assess systemic SCI-associated expression patterns and was not used as direct validation of central nervous system-resident microglial expression (Kyritsis et al., 2021).

### 2.12 Behavioral assessments

Neuromotor function was evaluated in both groups of mice (n = 8 per group) at baseline (pre-surgery) and at 1, 3, and 7 days post-injury. All experiments were conducted in a quiet environment by experimenters blinded to group allocation. (1) Rearing test: Mice were placed individually in a transparent cylinder and videotaped for 15 min. The first 20 climbing movements were analyzed, and the usage of the affected forelimb during rearing was quantified to assess spontaneous forelimb motor function (Schallert et al., 2000). (2) Grooming test: Spontaneous grooming behavior was induced by applying cool water, and the highest anatomical position reached by the forepaws during grooming was scored on a scale of 1–5 (Gensel et al., 2006). (3) Grip strength test: The maximum grasping force of both forepaws was measured using an electronic grip strength meter (DS2-20N). Each mouse was tested five times, and the mean of the three intermediate values was recorded. Data are presented as mean ± standard deviation (SD), and statistical analysis was performed using two-way repeated measures ANOVA.

### 2.13 Western blot analysis

Total protein was extracted from spinal cord tissues at the lesion site using radioimmunoprecipitation assay (RIPA) lysis buffer supplemented with protease and phosphatase inhibitors, and protein concentration was determined by the bicinchoninic acid (BCA) assay (Smith et al., 1985). Equal amounts of protein were separated by sodium dodecyl sulfate-polyacrylamide gel electrophoresis (SDS-PAGE) and subsequently transferred onto polyvinylidene difluoride (PVDF) membranes (Towbin et al., 1979). After blocking with 5% non-fat milk, the membranes were incubated overnight at 4 °C with primary antibodies against pan-Kla, ACSL4, VIM, IL-1β, and glyceraldehyde-3-phosphate dehydrogenase (GAPDH) (serving as an internal control). The following day, the membranes were incubated with horseradish peroxidase (HRP)-conjugated secondary antibodies, and signals were visualized using enhanced chemiluminescence. Densitometric quantification was performed using ImageJ software, and target protein expression levels were normalized to GAPDH (Schneider et al., 2012). Western blotting was performed using whole-lesion spinal cord tissues from biologically independent animals in the Sham and SCI 3 dpi groups (n = 4 per group). Therefore, Western blotting was interpreted as tissue-level protein validation reflecting multicellular lesion responses rather than microglia-specific protein changes. Detailed information on the primary and secondary antibodies used for Western blotting, including antibody name, catalog number, host species, dilution, manufacturer, target specificity, and application, is provided in Supplementary Table S2.

### 2.14 Immunofluorescence staining

Mice were deeply anesthetized and transcardially perfused with phosphate-buffered saline followed by 4% paraformaldehyde. Spinal cord tissues containing the C5 injury segment were harvested, post-fixed, dehydrated, embedded, and prepared as transverse frozen sections (Donaldson, 2001). Following blocking, sections were co-incubated overnight at 4 °C with primary antibodies against ionized calcium-binding adapter molecule 1 (IBA1) together with antibodies against VIM, pan-lysine lactylation (pan-Kla), or ACSL4. The following day, sections were incubated with species-specific fluorophore-conjugated secondary antibodies in the dark, and nuclei were counterstained with 4′,6-diamidino-2-phenylindole (DAPI). For anatomical standardization, immunofluorescence images were obtained from transverse C5 spinal cord sections. In SCI mice, sections centered on the C5 lesion epicenter were selected, whereas anatomically matched C5 sections were analyzed in Sham mice. The corresponding low-magnification anatomical context of the Sham and SCI lesion-center sections is shown by EC staining in Figure 7B. For each staining pair, representative fields were acquired from the selected C5 sections using the same acquisition parameters across groups. Fluorescence intensity of VIM, pan-Kla, or ACSL4 within IBA1-positive regions was quantified using ImageJ software. Measurements from representative fields were averaged to generate one biological value per animal before statistical analysis. Spatial overlap of target signals with IBA1-positive cells was evaluated qualitatively from merged images. Immunofluorescence analysis was performed using spinal cord sections from biologically independent animals in the Sham and SCI 3 dpi groups (n = 4 per group).

### 2.15 Statistical analysis

All statistical analyses were performed in the R environment (version 4.2.1) (R Core Team, 2022). Data visualization was performed using the ggplot2 package (Wickham, 2016). For bulk transcriptomic data, group comparisons and multiple-testing correction were mainly conducted using the limma package (Ritchie et al., 2015). Non-parametric multi-group comparisons were performed using the Kruskal–Wallis test, and correlations between continuous variables were assessed by Spearman or Pearson correlation coefficients. For single-cell data, spatiotemporal score differences were evaluated using linear mixed models (LMM) incorporating sample-level random effects (score ∼ group + (1 | sample)) or the Wilcoxon rank-sum test (Bates et al., 2015). For single-cell module-score comparisons, both cell-level visualization and sample-aware statistics were used. Cell-level distributions were shown for visualization, whereas linear mixed-effects models with sample as a random effect were applied where appropriate using the model score ∼ group + (1 | sample). Sample-level mean scores were also calculated as supplementary biological-replicate-level summaries. Wilcoxon rank-sum tests implemented in Seurat were used for exploratory marker-gene screening and subcluster annotation, and these cell-level differential expression results were interpreted together with bulk transcriptomic, network-based, and *in vivo* validation evidence. The statistical framework used for single-cell analyses is summarized in Supplementary Table S4. Data from animal experiments are presented as mean ± standard deviation (SD) for normally distributed variables. Comparisons between two groups were performed using independent-samples Student’s t-test, while comparisons among multiple groups were conducted using one-way ANOVA. For repeated-measures behavioral data across time points, two-way repeated-measures ANOVA (Two-way RM-ANOVA) was used, followed by post-hoc tests when appropriate. For immunofluorescence analyses, measurements from multiple representative fields of the same animal were averaged before statistical testing; therefore, each data point represented one biologically independent animal rather than an individual imaging field. A P value < 0.05 was considered statistically significant. Significance levels in figures are denoted as follows: ns, not significant (P ≥ 0.05); *P < 0.05; **P < 0.01; ***P < 0.001; ****P < 0.0001.

## 3 Results

### 3.1 Integration of bulk transcriptomic data reveals prominent time-dependent transcriptional remodeling after SCI

To investigate the potential interplay among lactylation, ferroptosis, and neuroinflammation at the tissue level following SCI, two bulk transcriptomic datasets (GSE5296 and GSE47681) were initially integrated (Wu et al., 2013; Faden and Hoffman, 2006).

**FIGURE 1:**
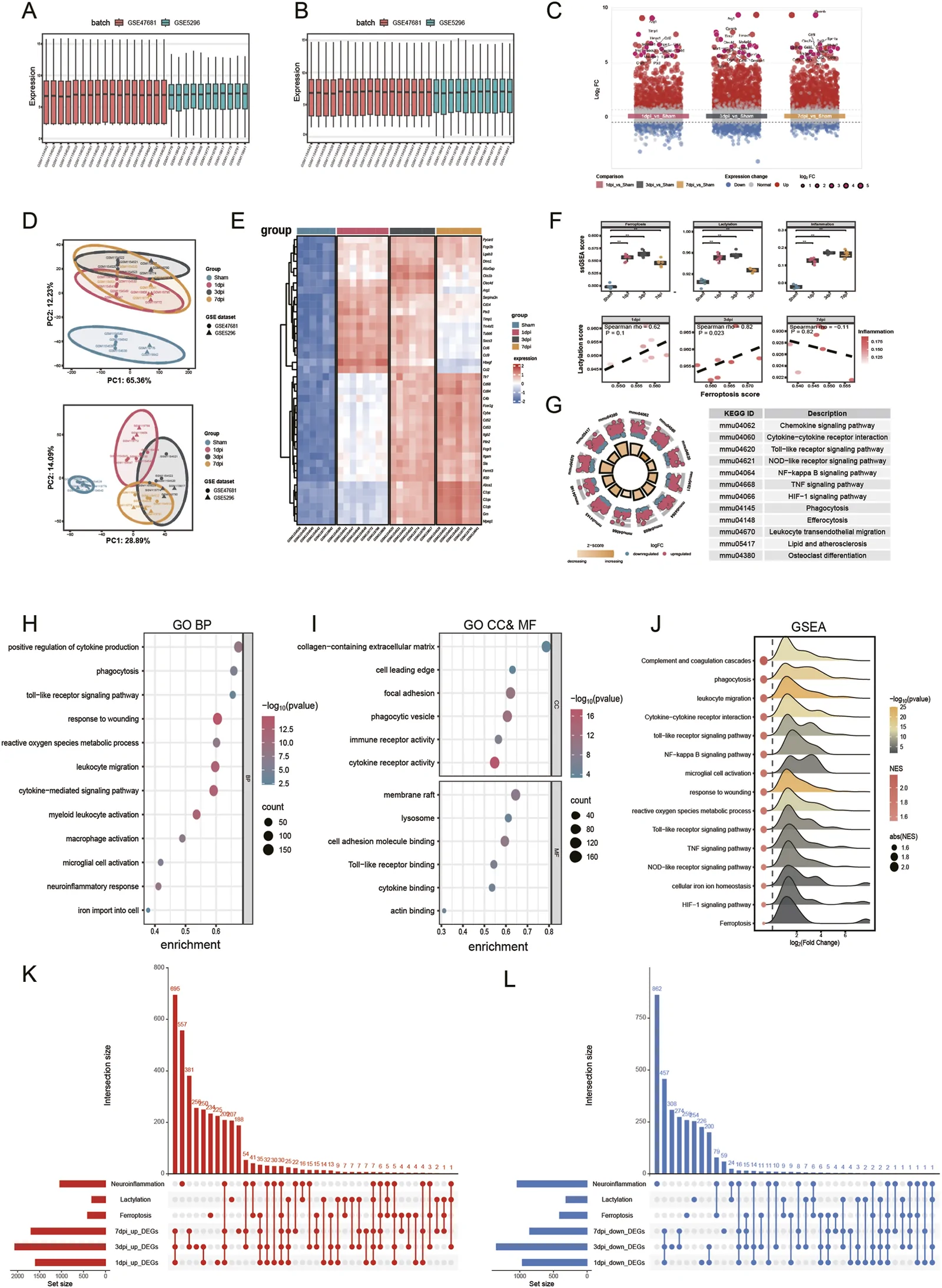
Bulk transcriptomic analysis reveals coordinated lactylation-related, ferroptosis-related, and inflammation-related signature changes after SCI. **(A, B)** Boxplots of sample expression distributions across multiple datasets before **(A)** and after **(B)** batch effect correction using the ComBat algorithm. **(C)** Volcano plots of differentially expressed genes (DEGs) in the 1 dpi, 3 dpi, and 7 dpi groups compared with the Sham group. **(D)** Principal component analysis (PCA) plots of samples before (top) and after (bottom) batch correction. **(E)** Unsupervised clustering heatmap of top DEG expression levels at different time points after SCI. **(F)** ssGSEA-derived lactylation-related, ferroptosis-related, and inflammation-related signature scores across time points, together with correlation trend plots among the three signature scores. **(G–I)** Pathway enrichment analysis of 3 dpi vs. Sham DEGs: **(G)** Circular plot of KEGG pathway enrichment, **(H)** Bubble plot of GO biological process (BP) terms, **(I)** Bubble plot of GO cellular component (CC) and molecular function (MF) terms. **(J)** Ridge plot of gene set enrichment analysis (GSEA) showing highly enriched immune- and metabolism-related pathways at 3 dpi. **(K,L)** UpSet plots of the intersections between upregulated DEGs **(K)** or downregulated DEGs **(L)** and the three functional gene sets.

Prior to batch effect correction, samples from different datasets exhibited a degree of separation in both expression distribution and PCA space, suggesting that dataset origin could exert systematic influence on the overall expression matrix. Following ComBat correction, the expression distributions across samples became more consistent, and PCA results demonstrated a marked attenuation of dataset-driven batch differences, while biological variation among different SCI time points was preserved (Figures 1A,B,D) (Leek et al., 2012). These findings indicated that the batch-corrected expression matrix was suitable for subsequent differential expression and functional scoring analyses.

Subsequently, differential expression profiles were compared for 1 dpi vs. Sham, 3 dpi vs. Sham, and 7 dpi vs. Sham, respectively. Multi-group volcano plots revealed 2,559 differentially expressed genes (DEGs) at 1 dpi (1,597 upregulated and 962 downregulated), 3,410 DEGs at 3 dpi (2,063 upregulated and 1,347 downregulated), and 2,549 DEGs at 7 dpi (1,696 upregulated and 853 downregulated), indicating extensive transcriptional remodeling in spinal cord tissue post-injury (Figure 1C). Notably, the most pronounced transcriptomic alterations were observed at 3 dpi, suggesting that this time point may represent a critical window of highly active secondary injury responses. A heatmap of top DEGs further demonstrated that samples from different time points could be distinctly segregated based on their differential gene expression patterns, confirming the stage-specific nature of transcriptional changes after SCI (Figure 1E).

### 3.2 Coordinated elevation of lactylation-related, ferroptosis-related, and inflammation-related signature scores after SCI

To evaluate the global changes in lactylation-, ferroptosis-, and inflammation-related programs, ssGSEA was performed on the batch-corrected bulk expression matrix using custom-defined lactylation-, ferroptosis-, and inflammation-related gene sets (Hänzelmann et al., 2013). The results demonstrated that, compared with the Sham group, the lactylation-related, ferroptosis-related, and inflammation-related signature scores were elevated to varying degrees at all post-SCI time points, with the 3 dpi group exhibiting the highest scores across all three categories (Figure 1F).

These observations suggest that SCI is associated with coordinated elevation of transcriptional signatures related to metabolic reprogramming, ferroptosis, and inflammatory responses. Correlation analysis within SCI samples revealed positive associations among the lactylation-related, ferroptosis-related, and inflammation-related signature scores, with the strongest association observed at 3 dpi (Spearman rho = 0.82, P = 0.023; Figure 1F). These findings indicate co-variation of the three transcriptional signature patterns at the subacute stage, but do not establish direct molecular coupling or causal regulation among these processes. Therefore, 3 dpi was selected as the focal time point for subsequent analyses of candidate genes and cell populations associated with these signatures.

### 3.3 DEGs at 3 dpi are predominantly enriched in pathways related to immune inflammation, phagocytosis, reactive oxygen species (ROS) metabolism, and ferroptosis

Given that the lactylation, ferroptosis, and inflammation scores all reached high levels and exhibited the strongest inter-correlation at 3 dpi, GO, KEGG, and GSEA enrichment analyses were further performed on the DEGs identified from the 3 dpi vs. Sham comparison (Yu et al., 2012; Subramanian et al., 2005). KEGG enrichment analysis revealed that the 3 dpi DEGs were significantly enriched in pathways including cytokine-cytokine receptor interaction, Toll-like receptor signaling pathway, TNF signaling pathway, NOD-like receptor signaling pathway, HIF-1 signaling pathway, phagosome, lipid and atherosclerosis, osteoclast differentiation, and ferroptosis (Figure 1G).

GO-BP analysis demonstrated that these DEGs were predominantly involved in biological processes such as positive regulation of cytokine production, phagocytosis, Toll-like receptor signaling pathway, response to wounding, reactive oxygen species metabolic process, leukocyte migration, cytokine-mediated signaling pathway, myeloid leukocyte activation, macrophage activation, microglial cell activation, neuroinflammatory response, and iron ion import into cell (Figure 1H). GO-CC and GO-MF analyses further indicated that the DEGs were closely associated with cellular components and molecular functions including collagen-containing extracellular matrix, phagocytic vesicle, lysosome, cell adhesion molecule binding, Toll-like receptor binding, cytokine binding, and actin binding (Figure 1I).

Consistently, GSEA results also demonstrated a positive enrichment trend for multiple immune-inflammatory and metabolism-related pathways following SCI, including complement and coagulation cascades, phagocytosis, leukocyte migration, cytokine-cytokine receptor interaction, Toll-like receptor signaling pathway, NF-kappa B signaling pathway, TNF signaling pathway, NOD-like receptor signaling pathway, HIF-1 signaling pathway, and ferroptosis (Figure 1J). Collectively, these results indicate that at 3 dpi, the injured spinal cord is characterized by a concomitantly active state of heightened inflammatory activation, myeloid cell responses, enhanced phagocytosis, oxidative stress, and ferroptosis-related programs.

### 3.4 Extensive overlap between time-point DEGs and lactylation, ferroptosis, and inflammation gene sets

To further clarify the relationship between DEGs at different time points and the three functional gene sets, the up- and downregulated DEGs at 1 dpi, 3 dpi, and 7 dpi were intersected with the lactylation, ferroptosis, and inflammation gene sets, respectively, and visualized using UpSet plots. The results showed that the upregulated DEGs exhibited the most pronounced overlap with the inflammation gene set, while also displaying substantial intersections with the ferroptosis and lactylation gene sets (Figure 1K). This indicates that the transcriptional programs upregulated after SCI encompass not only canonical inflammatory responses but also molecules associated with lactylation and ferroptosis.

UpSet analysis of downregulated DEGs also revealed that a subset of suppressed genes overlapped with the three functional gene sets; however, both the scale and functional implications of these intersections differed from those observed for upregulated genes (Figure 1L). Overall, the overlapping patterns of upregulated DEGs more strongly support the activation features of the coordinated lactylation-related, ferroptosis-related, and inflammation-related signature pattern following SCI and further suggest that these functional programs may share key regulatory genes.

### 3.5 Weighted gene co-expression network identifies hub genes associated with lactylation, ferroptosis, and inflammation

**FIGURE 2:**
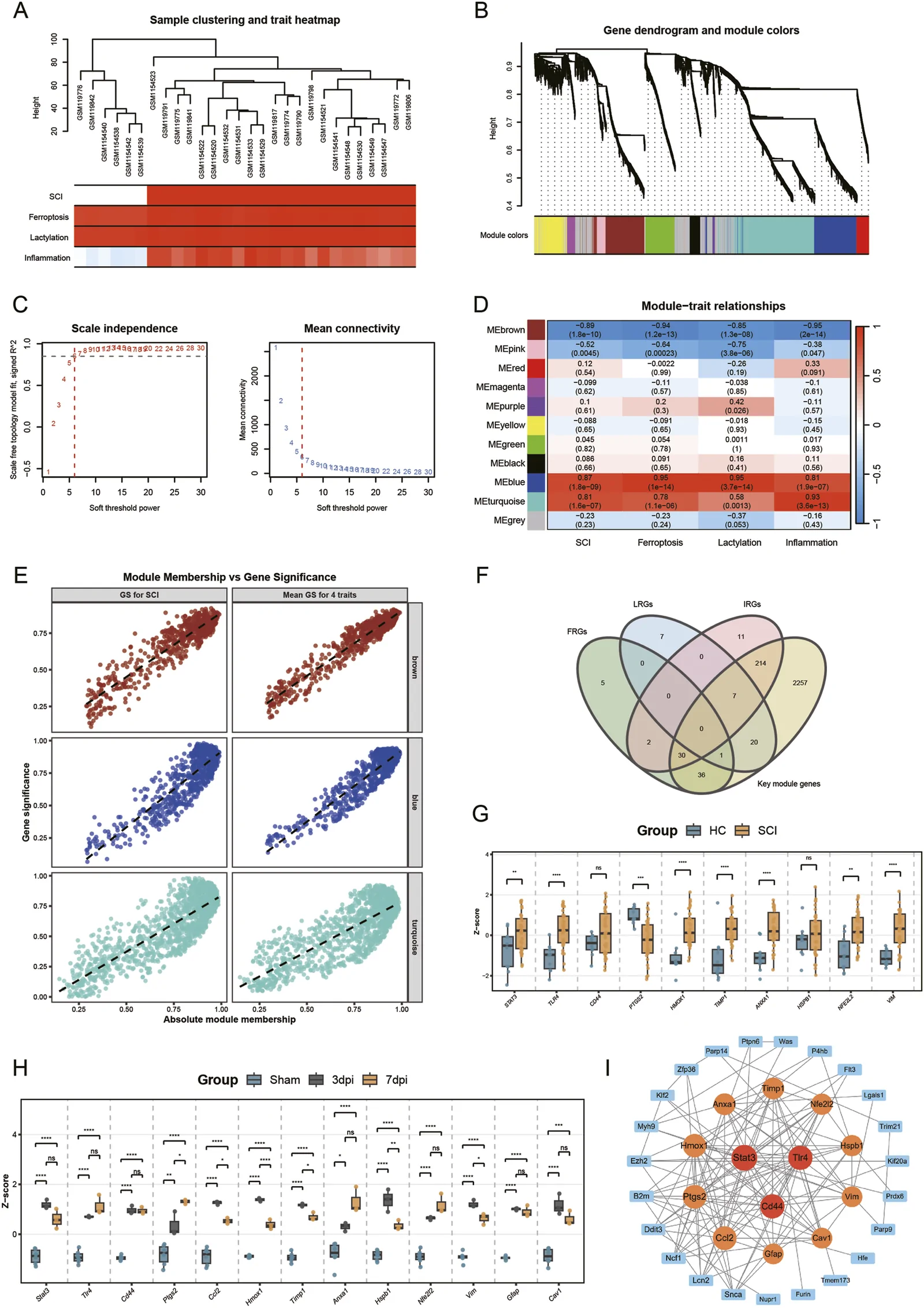
WGCNA and PPI network analysis prioritize candidate hub genes associated with SCI-related transcriptional signatures. **(A)** Sample clustering dendrogram with trait heatmap, including phenotypic traits (SCI status and the three module scores). **(B)** Gene co-expression network clustering dendrogram with module color assignment. **(C)** Soft-thresholding power screening for network topology analysis, displaying the scale-free fitting index and mean connectivity. **(D)** Module–trait correlation heatmap (numbers indicate correlation coefficients; values in parentheses indicate P values). **(E)** Scatter plots of module membership versus gene significance for genes within the key modules (brown, blue, and turquoise). **(F)** Venn diagram of the intersection among key module genes and lactylation-related genes (LRGs), ferroptosis-related genes (FRGs), and inflammation-related genes (IRGs). **(G)** Exploratory comparison of candidate hub-gene expression in peripheral blood leukocytes from healthy controls and patients with acute SCI in GSE151371. This analysis reflects systemic SCI-associated immune expression patterns and does not directly represent VIM expression in central nervous system-resident microglia. **(H)** Dynamic expression validation of the 13 candidate hub genes in the in-house mouse SCI transcriptomic sequencing cohort (Sham, 3 dpi, 7 dpi). **(I)** PPI network constructed from the 38 multi-intersection genes; 13 core hub genes were identified based on node degree. This analysis reflects systemic SCI-associated immune expression patterns and does not directly represent VIM expression in central nervous system-resident microglia.

To further identify gene modules associated with SCI status and the three functional scores at the systems-level co-expression network, weighted gene co-expression network analysis (WGCNA) was performed on the batch-corrected bulk expression matrix (Langfelder and Horvath, 2008). Sample clustering demonstrated an overall reasonable clustering without apparent outlier samples. Concurrently, the trait heatmap showed that SCI status, ferroptosis score, lactylation score, and inflammation score exhibited a pronounced increasing trend in SCI samples (Figure 2A). Soft-thresholding power screening indicated that when the power was set to 6, the network approximated a scale-free topology distribution while maintaining favorable mean connectivity; therefore, this parameter was selected to construct a signed co-expression network (Figure 2C). The gene clustering tree and module color assignment revealed that WGCNA identified a total of 10 co-expression modules (Figure 2B). Module-trait correlation analysis showed that the MEbrown module was significantly negatively correlated with SCI status and the three functional scores, whereas the MEblue and MEturquoise modules exhibited significant positive correlations with these traits (Figure 2D). Specifically, MEblue displayed higher correlations with ferroptosis and lactylation scores, while MEturquoise was more strongly correlated with the inflammation score, suggesting that these modules may represent distinct co-expression programs related to ferroptosis/lactylation and inflammation following SCI. Furthermore, scatter plots of module membership versus gene significance demonstrated good consistency between module affiliation and phenotypic relevance for genes within the brown, blue, and turquoise modules (Figure 2E). Consequently, the brown, blue, and turquoise modules were defined as the key modules for subsequent candidate gene screening.

### 3.6 Combined screening of hub genes via WGCNA, functional gene sets, and PPI network

To identify candidate genes with potential regulatory significance from the key modules, the brown, blue, and turquoise module genes were merged as key module genes and intersected with the lactylation, ferroptosis, and inflammation gene sets. The Venn diagram revealed that, although no gene was simultaneously shared by all four groups, a total of 38 candidate genes overlapped with the key module genes and at least two of the three functional gene sets (Figure 2F). These genes may be concurrently involved in co-expression module alterations and lactylation-, ferroptosis-, and inflammation-related transcriptional programs following SCI.

Subsequently, a protein–protein interaction (PPI) network was constructed based on the 38 candidate genes using the STRING database and Cytoscape software, and core nodes were screened with a degree cutoff > 10 (Szklarczyk et al., 2023; Shannon et al., 2003; Chin et al., 2014). Ultimately, 13 hub genes were identified, including Stat3, Tlr4, Cd44, Ptgs2, Ccl2, Hmox1, Timp1, Anxa1, Hspb1, Nfe2l2, Vim, Gfap, and Cav1 (Figure 2I). The majority of these genes are functionally associated with inflammatory signaling, oxidative stress, lipid metabolism, ferroptosis defense, glial activation, and cytoskeletal remodeling, suggesting their potential involvement in the lactylation-ferroptosis-inflammation-related pathological processes after SCI.

### 3.7 Independent mouse transcriptomic validation and exploratory human peripheral blood comparison support the SCI-relevance of hub genes

To validate the stability of the identified candidate hub genes in independent datasets, transcriptomic data from an in-house C5 cervical unilateral contusion SCI model were first analyzed. Compared with the Sham group, the majority of hub genes exhibited pronounced expression changes in the 3 dpi and 7 dpi groups, among which Stat3, Tlr4, Cd44, Ptgs2, Ccl2, Hmox1, Timp1, Anxa1, Nfe2l2, and Vim showed varying degrees of upregulation following SCI (Figure 2H). These results indicate that the candidate genes identified from public datasets demonstrate good reproducibility in an independent SCI model.

An exploratory external comparison was subsequently performed using the human SCI peripheral blood leukocyte RNA-seq dataset GSE151371 (Kyritsis et al., 2021). Among the detectable hub genes, several showed expression differences between healthy controls and patients with acute SCI that were directionally consistent with those observed in mouse SCI tissue (Figure 2G).

Importantly, because GSE151371 was derived from peripheral blood leukocytes rather than spinal cord tissue or cerebrospinal fluid, these findings should be interpreted as systemic SCI-associated immune expression patterns rather than direct validation of VIM expression in central nervous system-resident microglia. Thus, the peripheral blood analysis provides an orthogonal external reference for candidate hub genes but does not establish the central nervous system specificity of peripheral VIM.

### 3.8 Single-cell transcriptomic atlas identifies myeloid immune cells as major cell populations associated with SCI-related pathological signatures

**FIGURE 3:**
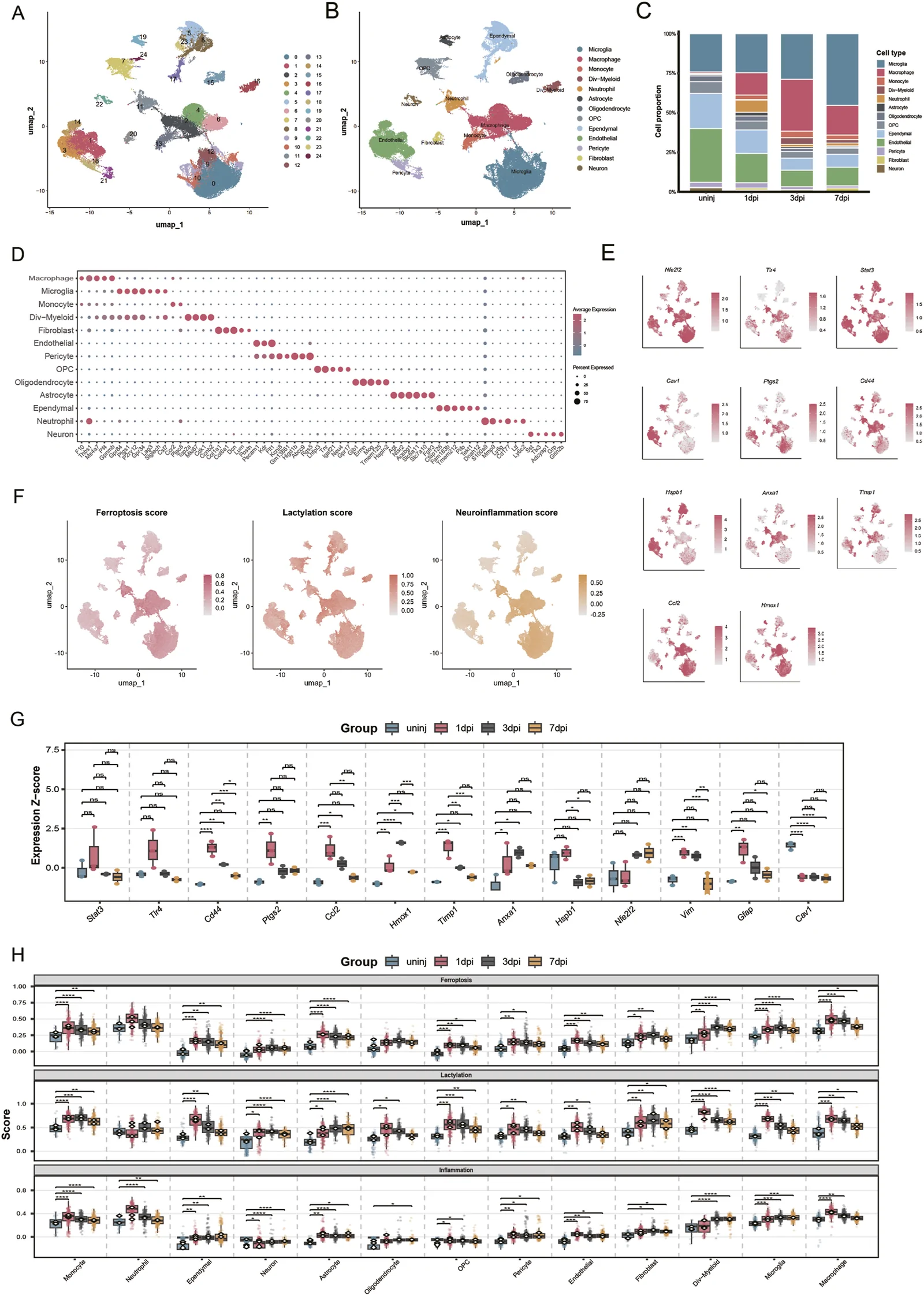
Single-cell transcriptomic atlas of spinal cord tissue after SCI and activation of pathological programs in microglia. **(A)** UMAP plot of all cells in the single-cell transcriptomic dataset, colored by cell type. **(B)** UMAP plot showing the spatial distribution of individual cell subpopulations. **(C)** Stacked bar plot of cell subpopulation proportions across different injury time points (Uninjured, 1 dpi, 3 dpi, 7 dpi). **(D)** Dot plot of the expression abundance of canonical marker genes used to identify major cell types. **(E)** Feature plots of key hub gene expression projected onto the UMAP space. **(F)** Feature plots of lactylation, ferroptosis, and inflammation functional scores mapped onto the UMAP space. **(G)** Boxplots comparing the expression levels of the 13 hub genes across different time points. **(H)** Boxplots comparing the lactylation, ferroptosis, and inflammation functional scores across different cell subpopulations.

To further delineate the cellular localization of lactylation-, ferroptosis-, and inflammation-related programs at single-cell resolution, a single-cell transcriptomic atlas of the injured spinal cord was constructed using the GSE162610 dataset (Milich et al., 2021). Following quality control, batch correction, and unsupervised clustering, a total of 25 cell clusters were identified (Figure 3A). Based on canonical marker gene expression, these clusters were subsequently annotated into 13 major cell types: Microglia, Macrophage, Monocyte, Div-Myeloid, Neutrophil, Astrocyte, Oligodendrocyte, OPC, Ependymal, Endothelial, Pericyte, Fibroblast, and Neuron (Figure 3B). The expression patterns of classical marker genes across cell populations were consistent with established profiles, confirming the accuracy of the annotation (Figure 3D). Temporal analysis of cell composition proportions revealed that, compared with the uninjured group, the proportions of immune cells—particularly macrophages and monocytes—rapidly expanded after SCI, indicating that the injury microenvironment elicits a robust myeloid cell infiltration and proliferation response (Figure 3C).

To identify the cell populations in which the core transcriptional programs derived from the preceding bulk analyses were activated, the three functional module scores and the candidate hub genes were projected onto the UMAP space (Hao et al., 2021). Feature plots intuitively demonstrated that the module scores for lactylation, ferroptosis, and neuroinflammation exhibited highly similar spatial distributions within the single-cell landscape, with the overwhelming majority of high-scoring signals specifically concentrated in regions corresponding to microglia and macrophages (Figure 3F). Moreover, the previously identified candidate hub genes (e.g., Stat3, Tlr4, Cd44, Ptgs2, Ccl2, Hmox1, Timp1, Anxa1, Nfe2l2, and Vim) were also predominantly enriched in myeloid immune cell populations (Figure 3E).

On this basis, the dynamic temporal expression trends of the 13 candidate hub genes following SCI were further quantitatively assessed. Box plot results demonstrated that genes such as Ptgs2, Ccl2, Hmox1, Timp1, Anxa1, Nfe2l2, Vim, Gfap, and Cav1 exhibited significant time-dependent upregulation during the early or subacute phase (1–3 dpi) after injury (Figure 3G). Subsequently, the AddModuleScore function was employed to calculate the lactylation, ferroptosis, and inflammation scores for each cell type at the single-cell level. The results showed that the three scores in Microglia and Macrophage were all significantly elevated post-SCI, particularly at 1 dpi and 3 dpi, whereas the score changes in other cell types were relatively modest (Figure 3H). This distribution pattern was consistent with the bulk-level finding that the three scores peaked at 3 dpi, suggesting that the coordinated elevation of lactylation-related, ferroptosis-related, and inflammatory signature scores following SCI is mainly associated with myeloid immune cells. Among them, microglia, as resident innate immune cells of the central nervous system, represented a major cell population associated with these transcriptional signatures. These findings prompted us to further focus on microglia for refined subpopulation analysis.

### 3.9 Microglial subclustering reveals expansion of pathological DAM-like states after SCI

**FIGURE 4:**
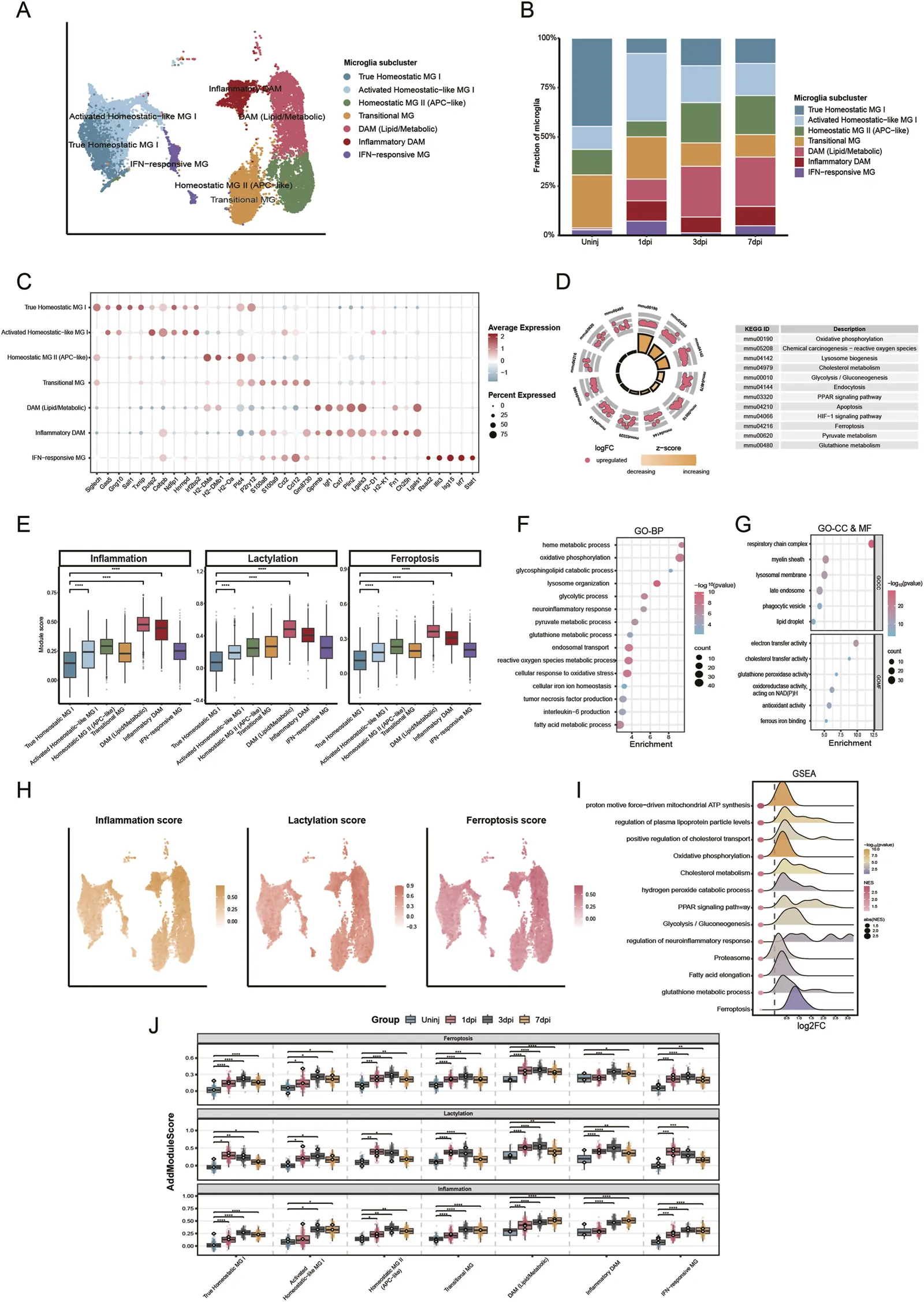
Microglial heterogeneity and identification of disease-associated microglia (DAM) subpopulations under pathological conditions. **(A)** UMAP plot of seven refined microglial subclusters. **(B)** Stacked bar plot showing the proportional changes of each microglial subpopulation across time points. **(C)** Dot plot of signature gene expression for the seven microglial subpopulations. **(D)** Circular KEGG enrichment plot of signature genes of pathological DAM-like microglia (combined DAM). **(E)** Boxplots comparing the lactylation, ferroptosis, and inflammation scores among the seven microglial subpopulations. **(F,G)** Bubble plots of GO-BP **(F)** and GO-CC/MF **(G)** enrichment analyses of combined DAM signature genes. **(H)** Feature plots of inflammation, lactylation, and ferroptosis scores projected onto the microglial UMAP space. **(I)** GSEA plots of pathways significantly enriched in combined DAM cells. **(J)** Dynamic changes in inflammation, lactylation, and ferroptosis scores of each microglial subpopulation at different post-injury time points.

To further resolve the heterogeneity within the microglial population, microglia were extracted from the global single-cell dataset and subjected to a second round of clustering. Based on marker genes and functional characteristics, seven microglial subclusters were ultimately identified: True Homeostatic MG I, Activated Homeostatic-like MG I, Homeostatic MG II APC-like, Transitional MG, DAM lipid/metabolic, Inflammatory DAM, and IFN-responsive MG (Figures 4A,C) (Keren-Shaul et al., 2017; Masuda et al., 2019).

Analysis of subpopulation proportions revealed that homeostatic microglia predominated in the uninjured group, whereas a pronounced shift in microglial composition was observed after SCI. As injury progressed, the proportions of DAM lipid/metabolic and Inflammatory DAM gradually increased, particularly at 3 dpi and 7 dpi (Figure 4B). These results indicate that microglia undergo a transition from a homeostatic-like state toward DAM-like and inflammation-associated pathological states following SCI (Keren-Shaul et al., 2017).

Based on their functional profiles, the DAM lipid/metabolic and Inflammatory DAM subpopulations were collectively defined as pathological DAM-like microglia, designated combined DAM. KEGG analysis of combined DAM marker genes demonstrated significant enrichment in pathways including oxidative phosphorylation, chemical carcinogenesis-reactive oxygen species, lysosome, cholesterol metabolism, glycolysis/gluconeogenesis, PPAR signaling pathway, ferroptosis, pyruvate metabolism, and glutathione metabolism (Figure 4D) (Yu et al., 2012). These pathways are heavily implicated in metabolic reprogramming, oxidative stress, lipid metabolism, and ferroptosis, suggesting that combined DAM represents a major pathological microglial state associated with concurrent lactylation-related, ferroptosis-related, and inflammation-related signatures after SCI.

### 3.10 Pathological microglia exhibit elevated lactylation, ferroptosis, and inflammation scores

To further validate the functional status of distinct microglial subpopulations, the lactylation, ferroptosis, and inflammation scores were compared across the seven microglial subclusters. The results demonstrated that the three scores in DAM lipid/metabolic and Inflammatory DAM were overall significantly higher than those in homeostatic microglial subpopulations, indicating that pathological DAM-like microglia concurrently harbor metabolic reprogramming, ferroptosis-related, and inflammatory activation features (Figure 4E).

GO-BP analysis revealed that combined DAM marker genes were predominantly enriched in biological processes such as heme metabolic process, oxidative phosphorylation, glycolipid catabolic process, lysosome organization, glycolytic process, neuroinflammatory response, pyruvate metabolic process, glutathione metabolic process, reactive oxygen species metabolic process, cellular response to oxidative stress, cellular iron ion homeostasis, tumor necrosis factor production, interleukin-6 production, and fatty acid metabolic process (Figure 4F) (Yu et al., 2012). GO-CC and GO-MF analyses further indicated that these genes were closely associated with cellular components and molecular functions including respiratory chain complex, myelin sheath, lysosomal membrane, late endosome, phagocytic vesicle, cholesterol transfer activity, glutathione peroxidase activity, oxidoreductase activity, and ferrous iron binding (Figure 4G) (Yu et al., 2012).

UMAP projection showed that the lactylation, ferroptosis, and inflammation scores exhibited similar distributions within the microglial space and were predominantly concentrated in regions occupied by DAM lipid/metabolic and Inflammatory DAM (Figure 4H). GSEA results further supported the enrichment of pathways such as proton motive force-driven mitochondrial ATP synthesis, regulation of plasma lipoprotein particle levels, cholesterol metabolism, oxidative phosphorylation, PPAR signaling pathway, glycolysis/gluconeogenesis, glutathione metabolic process, and ferroptosis in pathological DAM-like microglia (Figure 4I) (Subramanian et al., 2005). Time-stratified scoring analysis also demonstrated that DAM lipid/metabolic and Inflammatory DAM maintained persistently elevated module scores after SCI (Figure 4J). Collectively, these results indicate that pathological DAM-like microglial states are major microglial populations associated with concurrent lactylation-related, ferroptosis-related, and inflammatory transcriptional signatures after SCI.

### 3.11 Pseudotime trajectory analysis suggests an inferred transcriptional continuum from homeostatic-like to pathologically activated microglial states

**FIGURE 5:**
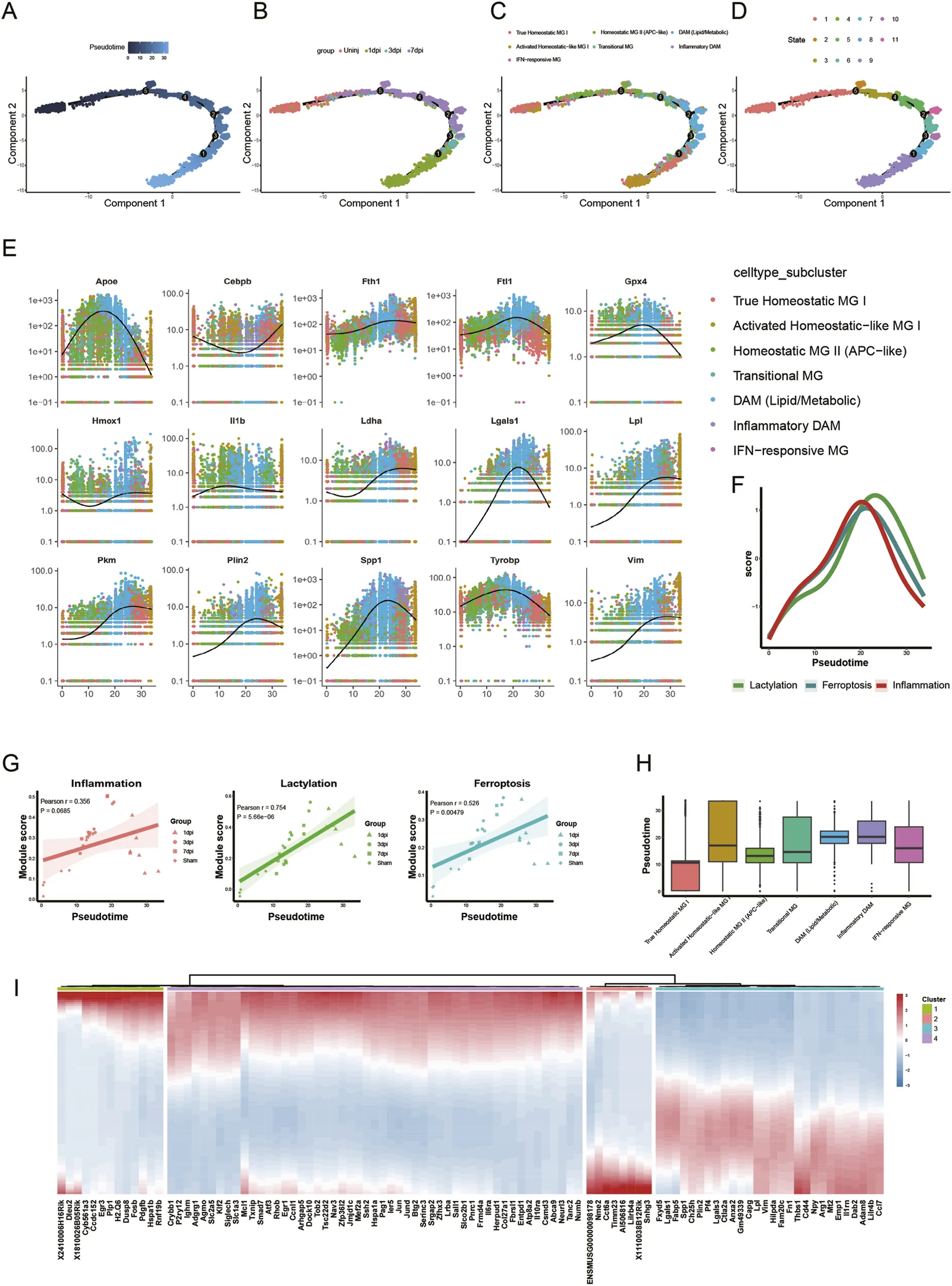
Pseudotime trajectory reconstruction of the continuous transition of microglia from a homeostatic to a pathological state. **(A–D)** Monocle2 pseudotime trajectory plots, with cells colored by pseudotime value **(A)**, injury time group **(B)**, microglial subcluster classification **(C)**, and differentiation state **(D)**. **(E)** Dynamic fitting curves of key genes (e.g., Apoe, Hmox1, Il1b, Lgals1, Plin2, Spp1, Tyrobp, and Vim) along the pseudotime trajectory. **(F)** Trend line plots of lactylation, ferroptosis, and inflammation module scores along the pseudotime trajectory. **(G)** Correlation scatter plots between the three functional scores and pseudotime. **(H)** Boxplots of the pseudotime distribution of each microglial subpopulation. **(I)** Heatmap of the top 50 pseudotime-associated genes, illustrating gene network switching during cell state transition.

To further delineate the microglial state transition process following SCI, pseudotime trajectory analysis was performed on microglia using Monocle2 (Qiu et al., 2017). The results revealed an inferred transcriptional continuum of microglial states along pseudotime, extending from homeostatic-like to pathologically activated states. With True Homeostatic MG I from the Sham group set as the root, the initial segment of the trajectory was predominantly enriched with homeostatic microglia, whereas Transitional MG, DAM lipid/metabolic, Inflammatory DAM, and IFN-responsive MG progressively emerged in the mid-to-late segments of the pseudotime trajectory (Figures 5A–D) (Keren-Shaul et al., 2017).

The distribution of different time points along the pseudotime trajectory further demonstrated that cells from the Sham group were primarily located at the early pseudotime stage, whereas cells from post-SCI time points progressively shifted toward the mid-to-late stages, consistent with injury-associated redistribution of microglial transcriptional states toward later pseudotime positions (Figure 5B). The pseudotime distribution across subpopulations also showed that True Homeostatic MG I was positioned early in the trajectory, while DAM lipid/metabolic and Inflammatory DAM were more concentrated in the mid-to-late stages (Figure 5H).

Dynamic expression analysis of key genes along the pseudotime trajectory revealed that Apoe, Lgals1, Lpl, Plin2, Spp1, Tyrobp, Il1b, Ldha, Hmox1, and Vim gradually increased or exhibited phased peaks as pseudotime progressed. Iron metabolism-related genes such as Gpx4, Fth1, and Ftl1 also displayed dynamic changes (Figure 5E). The lactylation, ferroptosis, and inflammation module scores exhibited synchronous or phased enhancement trends along the pseudotime trajectory (Figure 5F) and were positively correlated with pseudotime (Figure 5G). A heatmap of the top 50 pseudotime-associated genes further highlighted pronounced transcriptional module remodeling during the microglial state transition (Figure 5I).

Together, these results suggest that pseudotime analysis captures an inferred transcriptional continuum rather than experimentally observed lineage conversion. Homeostatic-like microglia were enriched at early pseudotime positions, whereas transitional, DAM lipid/metabolic, inflammatory DAM, and IFN-responsive states were enriched at later positions. Lactylation-related, ferroptosis-related, and inflammation-related signature scores increased along this inferred continuum, supporting their association with pathological microglial state remodeling after SCI.

### 3.12 VIM Is Prioritized as a candidate hub gene associated with lactylation-related, ferroptosis-related, and inflammation-related signature scores

**FIGURE 6:**
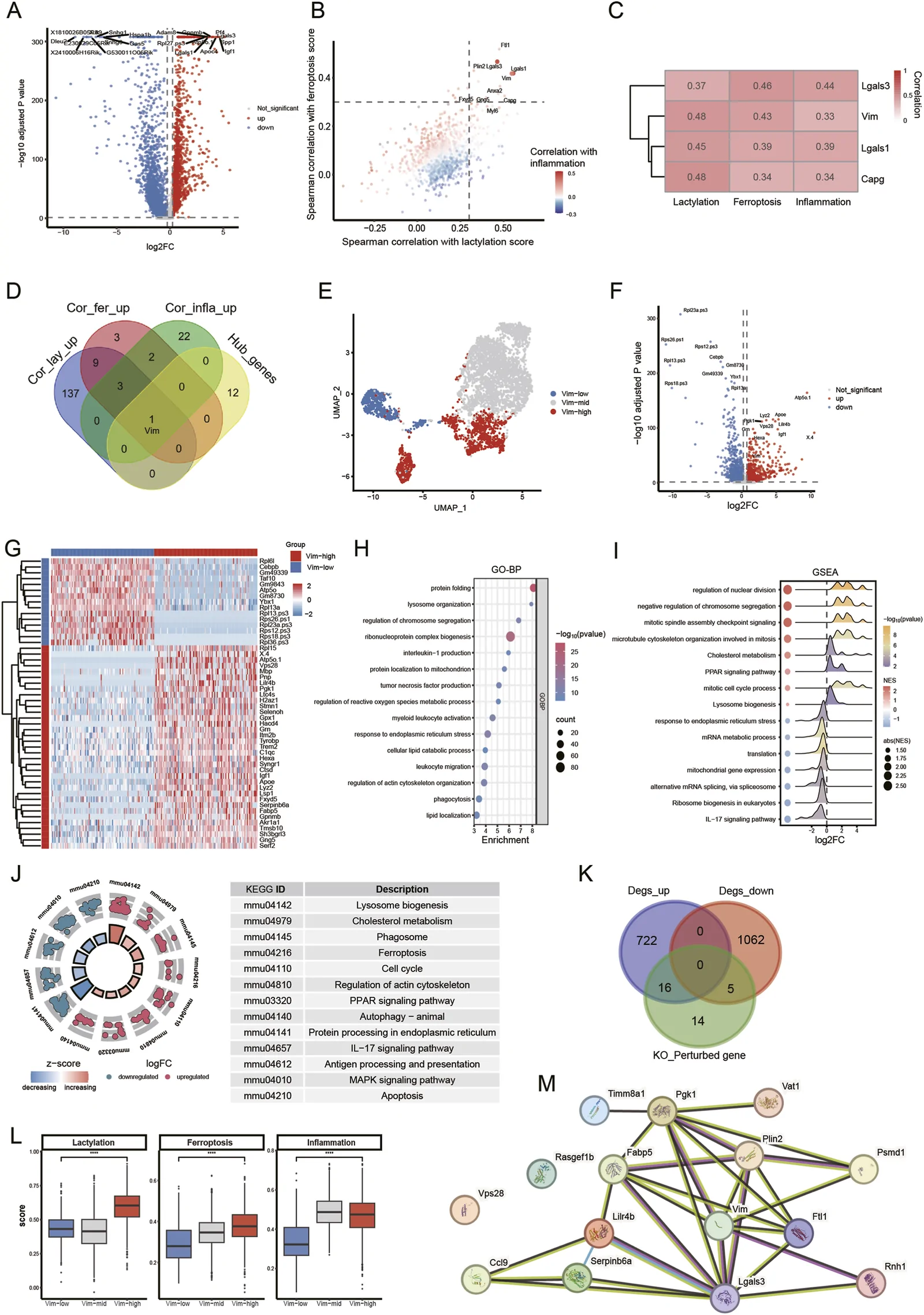
Prioritization of Vim as a candidate hub associated with pathological DAM-like microglial states. **(A)** Exploratory marker-gene screening between combined DAM and True Homeostatic MG I. **(B,C)** Correlation analysis between candidate genes and lactylation-related, ferroptosis-related, and neuroinflammation-related signature scores in combined DAM cells. **(D)** Intersection of triple-score-associated genes with hub genes identified by the bulk WGCNA-PPI pipeline, prioritizing Vim as a candidate hub gene. **(E)** Re-clustering of non-Sham combined DAM cells and operational stratification into Vim-low, Vim-mid, and Vim-high groups based on cluster-level median Vim expression. **(F,G)** Exploratory cell-level DEG analysis comparing Vim-high and Vim-low clusters. **(H–J)** GO, GSEA, and KEGG analyses of genes associated with the Vim-high transcriptional state. **(K)** Intersection between exploratory Vim-high versus Vim-low DEGs and scTenifoldKnk-predicted Vim perturbation-associated genes. **(L)** Comparison of lactylation-related, ferroptosis-related, and neuroinflammation-related signature scores among Vim-low, Vim-mid, and Vim-high groups. **(M)** PPI network of overlapping candidate VIM-associated genes predicted by *in silico* perturbation analysis. Cell-level DEG analyses in this figure were used for exploratory functional annotation. The scTenifoldKnk analysis was interpreted as hypothesis-generating *in silico* perturbation rather than experimental Vim loss-of-function validation.

Exploratory cell-level differential expression analysis between combined DAM and True Homeostatic MG I was first performed using the Wilcoxon rank-sum test in Seurat to support marker-gene nomination and downstream functional annotation (Hao et al., 2021). The volcano plot revealed that, compared with homeostatic microglia, a large number of genes were significantly upregulated in combined DAM, including pathology-associated genes such as Vim, Lgals3, Lgals1, Plin2, Apoe, Spp1, Ccl2, Il1b, and Capg (Figure 6A).

Subsequently, Spearman correlations between the expression levels of the upregulated genes and the lactylation, ferroptosis, and inflammation scores were calculated within the combined DAM cells. The correlation scatter plot demonstrated that only a subset of the upregulated genes in combined DAM were simultaneously positively correlated with all three scores (Figure 6B). Candidate genes were further filtered by requiring a Spearman correlation coefficient ≥ 0.30 and an adjusted P value < 0.05 with all three scores. The resulting heatmap showed that Lgals3, Vim, Lgals1, and Capg exhibited high correlations with the three scores (Figure 6C).

Intersecting these triple-score-correlated genes with the hub genes previously identified through the bulk-WGCNA-PPI pipeline (Szklarczyk et al., 2023; Shannon et al., 2003; Chin et al., 2014) ultimately revealed Vim as the only candidate gene concurrently present in the lactylation-related, ferroptosis-related, inflammation-related, and hub gene sets (Figure 6D). This finding prioritizes Vim as a candidate hub gene associated with lactylation-related, ferroptosis-related, and inflammation-related signature scores in pathological microglia following SCI.

### 3.13 Vim-high pathological microglia exhibit an activated inflammatory-metabolic transcriptional state

To further characterize transcriptional states associated with Vim expression in pathological microglia, combined DAM cells from non-Sham groups were extracted and subjected to re-clustering and stratification based on Vim expression levels (Hao et al., 2021). According to the median Vim expression of each cluster, the pathological microglia were categorized into Vim-low, Vim-mid, and Vim-high groups (Figure 6E).

Compared with Vim-low cells, a substantial number of genes were differentially expressed in Vim-high pathological microglia. The volcano plot and heatmap revealed that upregulated genes in Vim-high cells included a variety of inflammation-, phagocytosis-, lysosome-, lipid metabolism-, and stress-associated molecules, whereas Vim-low cells retained relatively weaker pathological activation features (Figures 6F,G).

GO-BP enrichment analysis demonstrated that the Vim-high vs. Vim-low DEGs were predominantly involved in biological processes such as proton folding, lysosome organization, regulation of chromosome segregation, ribonucleoprotein complex biogenesis, protein localization to mitochondrion, regulation of reactive oxygen species metabolic process, myeloid leukocyte activation, response to endoplasmic reticulum stress, cellular lipid catabolic process, leukocyte migration, regulation of actin cytoskeleton organization, phagocytosis, and lipid localization (Figure 6H) (Yu et al., 2012). GSEA results further indicated that Vim-high cells were enriched for pathways including regulation of nuclear division, mitotic spindle assembly checkpoint signaling, microtubule cytoskeleton organization, cholesterol metabolism, PPAR signaling pathway, myeloid cell response, lysosome biogenesis, mRNA metabolic process, mitochondrial gene expression, ribosome biogenesis in eukaryotes, and IL-17 signaling pathway (Figure 6I) (Subramanian et al., 2005).

KEGG analysis showed that genes associated with Vim-high pathological microglia were enriched in lysosome biogenesis, cholesterol metabolism, phagosome, ferroptosis, regulation of actin cytoskeleton, PPAR signaling pathway, autophagy, protein processing in endoplasmic reticulum, IL-17 signaling pathway, antigen processing and presentation, MAPK signaling pathway, and apoptosis (Figure 6J) (Yu et al., 2012). These results indicate that Vim-high pathological microglia exhibit an activated transcriptional state enriched for inflammatory, lipid-metabolic, lysosomal, phagocytic, ferroptosis-related, and cytoskeletal-remodeling programs.

Further comparison of module scores among Vim-low, Vim-mid, and Vim-high cells demonstrated that the lactylation, ferroptosis, and inflammation scores in the Vim-high group were all significantly higher than those in the Vim-low and Vim-mid groups (Figure 6L) (Hao et al., 2021). This observation further supports an association between higher Vim expression and elevated lactylation-related, ferroptosis-related, and inflammation-related signature scores within pathological microglia.

### 3.14 *In silico* vim perturbation predicts candidate VIM-associated network changes in pathological microglia

To explore putative VIM-associated network changes in pathological microglia, *in silico* Vim perturbation analysis was performed using scTenifoldKnk. This analysis predicted a set of Vim perturbation-associated genes. Intersection analysis between these predicted perturbation-associated genes and the exploratory Vim-high versus Vim-low DEGs identified overlapping candidates associated with Vim expression stratification (Figure 6K).

The PPI network of the overlapping candidates included molecules such as Lgals3, Ftl1, Plin2, Fabp5, Pgk1, Timp1, and Hspb1, which are implicated in lipid metabolism, iron homeostasis, glycolysis, stress responses, and inflammatory activation (Figure 6M). These *in silico* findings suggest that VIM-associated network changes may involve metabolic adaptation, lysosomal/phagocytic activity, lipid processing, and ferroptosis-related transcriptional programs. However, these predictions do not establish VIM-dependent regulation and require direct functional validation.

### 3.15 Validation of post-SCI motor deficits and upregulation of VIM, pan-Kla, ACSL4, and IL-1β in a C5 contusion SCI mouse model

**FIGURE 7:**
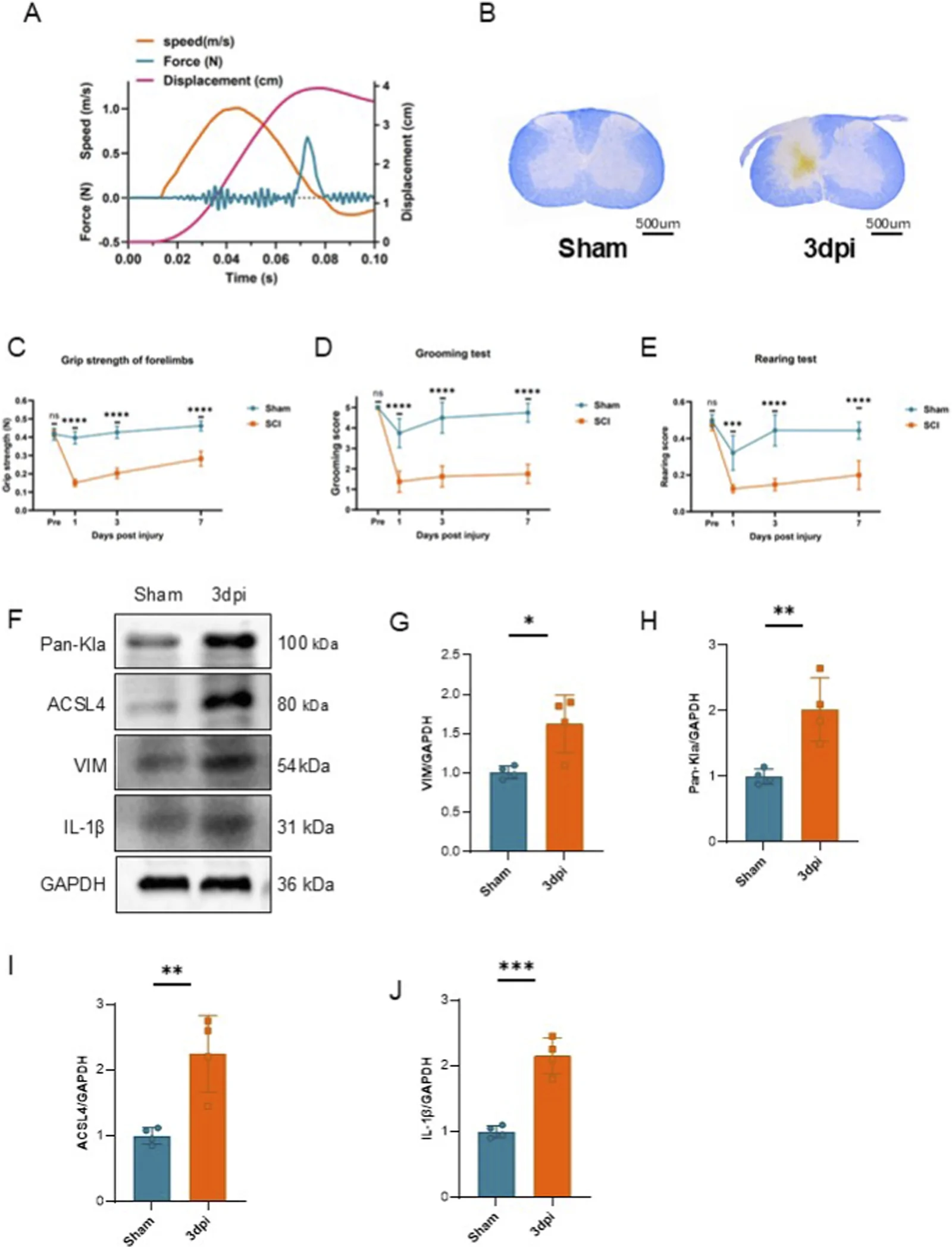
Establishment of the C5 unilateral contusion SCI model and whole-lesion protein validation. **(A)** Biomechanical parameter curves (velocity, force, and displacement over time) recorded in real time during contusion injury using the spinal cord impactor. **(B)** Representative EC-stained transverse C5 spinal cord sections from Sham and SCI 3 dpi mice, showing lesion-center tissue disruption in the injured hemicord. These sections provide low-magnification anatomical context for the lesion-centered immunofluorescence analyses shown in Figure 8. **(C–E)** Temporal change curves of forelimb grip strength, grooming scores, and rearing scores (n = 8 per group; mean ± SD; *, P < 0.05; **, P < 0.01; ***, P < 0.001). **(F)** Western blot analysis and quantification of VIM, pan-Kla, ACSL4, and IL-1β in whole-lesion spinal cord tissue at 3 dpi. **(G–J)** Semi-quantitative bar plots comparing the relative expression levels of the above target proteins after normalization to the internal control (n = 4 per group; mean ± SD; *P < 0.05, **P < 0.01, ***P < 0.001). These Western blot data should be interpreted as multicellular lesion-level protein responses rather than microglia-specific protein changes.

To further validate the bioinformatic findings, an adult male C57BL/6J mouse model of C5 unilateral contusion SCI was established (Huang et al., 2019). Recording of injury parameters demonstrated stable impact velocity, force, and displacement during the SCI modeling procedure, indicating good consistency in model generation (Figure 7A). Histological observation of spinal cord cross-sections revealed pronounced tissue architecture disruption at the injury epicenter in the SCI 3 dpi group compared with the Sham group, confirming successful establishment of the C5 unilateral contusion model (Figure 7B).

Behavioral experiments were subsequently performed to evaluate changes in forelimb motor function after SCI. Compared with the Sham group, mice in the SCI group exhibited significantly decreased forelimb grip strength at 1, 3, and 7 dpi, indicating impaired forelimb muscle strength following cervical SCI (Figure 7C). The grooming test results demonstrated that post-injury grooming scores were markedly reduced in SCI mice, reflecting impaired fine motor function of the forelimb (Figure 7D) (Gensel et al., 2006). Furthermore, the rearing test revealed a pronounced reduction in the use of the affected forelimb in SCI mice, indicating impaired spontaneous forelimb motor function after injury (Figure 7E) (Schallert et al., 2000). Collectively, these results demonstrate that this C5 unilateral contusion model consistently induces forelimb motor dysfunction following cervical SCI.

Western blot analysis was subsequently performed using whole-lesion spinal cord tissues at the injury epicenter at 3 dpi (Towbin et al., 1979). The results showed that VIM protein expression was markedly upregulated in the SCI 3 dpi group compared with the Sham group, suggesting increased lesion-level cytoskeletal and glial-reactive responses after SCI. Concurrently, pan-Kla levels were elevated, indicating enhanced global protein lactylation signals at the tissue level; ACSL4 expression was increased, suggesting activation of ferroptosis-related lipid metabolic programs; and IL-1β expression was upregulated, indicating enhanced inflammatory responses (Figures 7F–J). Because these Western blot assays were performed using whole-lesion tissue lysates, the results reflect multicellular tissue-level responses and should not be interpreted as microglia-specific protein changes.

**FIGURE 8:**
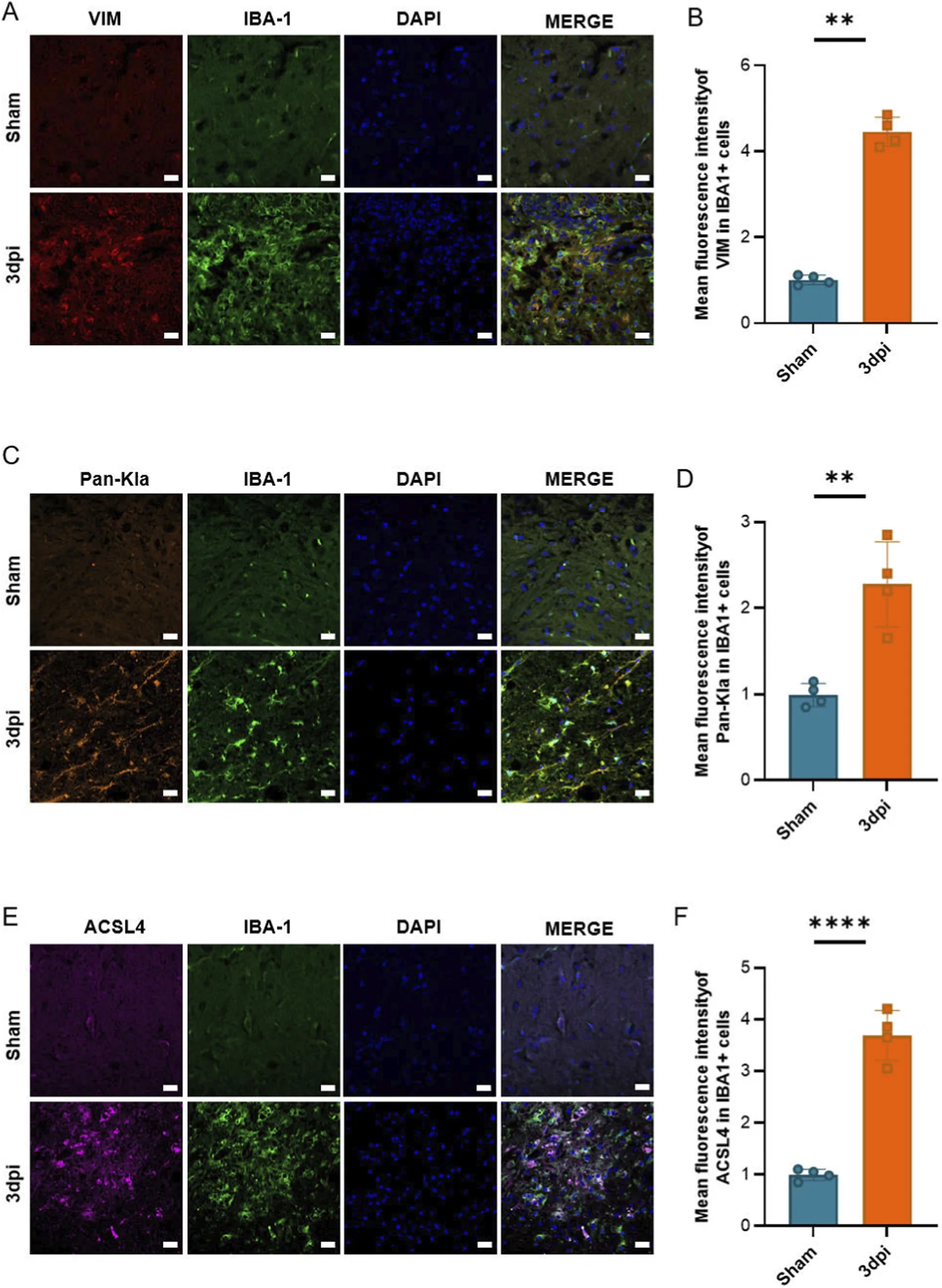
Immunofluorescence assessment of VIM, pan-Kla, and ACSL4 signals within IBA1-positive cells after C5 spinal cord injury. **(A,C,E)** Representative *in situ* immunofluorescence double-staining images showing IBA1 (green) together with VIM (red, **(A)**, pan-lysine lactylation (pan-Kla; orange, **(C)**, or ACSL4 (purple, **(E)**. Nuclei were counterstained with DAPI (blue). Representative images were obtained from transverse C5 spinal cord sections. In SCI mice, lesion-center sections were analyzed, whereas anatomically matched C5 sections were evaluated in Sham mice. The corresponding low-magnification EC-stained anatomical context is provided in Figure 7B. Scale bars: 20 μm. **(B,D,F)** Quantification of mean fluorescence intensity of VIM **(B)**, pan-Kla **(D)**, and ACSL4 **(F)** within IBA1-positive regions in Sham and SCI 3 dpi spinal cord sections. Measurements from representative fields were averaged to generate one biological value per animal. Data are presented as mean ± SD; n = 4 biologically independent animals per group. Statistical comparisons were performed using two-tailed unpaired Student’s t-tests. *P < 0.05; **P < 0.01; ****P < 0.0001.

Immunofluorescence analyses were performed on transverse C5 spinal cord sections. In SCI mice, lesion-center sections were selected, whereas anatomically matched C5 sections were analyzed in Sham mice; the corresponding low-magnification EC-stained anatomical context is shown in Figure 7B. At 3 dpi, VIM, pan-Kla, and ACSL4 signals showed increased spatial overlap with IBA1-positive cells in the SCI group compared with the Sham group. Compared with the Sham group, the fluorescence intensity of VIM (Figures 8A,B), pan-Kla (Figures 8C,D), and ACSL4 (Figures 8E,F) within IBA1-positive regions was significantly increased in the SCI group.

These results are consistent with the bulk transcriptomic and single-cell RNA-seq analyses, supporting the association of a VIM-associated IBA1-positive pathological myeloid-cell state with concurrent lactylation-related, ferroptosis-related, and inflammatory signatures after SCI.

## 4 Discussion

The pathological mechanisms underlying secondary injury after SCI are highly intricate, wherein immune-cell infiltration, metabolic reprogramming, oxidative stress, and programmed cell death collectively shape the post-injury microenvironment and impede neurological recovery (Ahuja et al., 2017). Previous studies have shown that glycolysis-associated lactylation and iron-dependent lipid peroxidation, termed ferroptosis, are involved in inflammatory activation and secondary tissue damage after SCI (Zhang et al., 2019; Dixon et al., 2012). However, whether lactylation-related, ferroptosis-related, and inflammatory transcriptional signatures show coordinated changes after SCI, and which microglial states are associated with these signatures at single-cell resolution, remain incompletely understood.

To address this question, the present study integrated multi-cohort bulk transcriptomic data, single-cell RNA sequencing (scRNA-seq), co-expression network analysis, *in silico* perturbation analysis, and *in vivo* validation. The analyses identified a coordinated pattern of lactylation-related, ferroptosis-related, and inflammation-related transcriptional signatures that peaked at 3 dpi. These signatures were predominantly associated with pathological disease-associated microglia (DAM)-like states, and Vim was prioritized as a candidate hub gene within this transcriptional framework. Collectively, these findings provide a transcriptomic perspective on metabolic, ferroptosis-related, and inflammatory state remodeling during secondary SCI. The secondary injury cascade following SCI is highly time-dependent. The tissue-level bulk transcriptomic analysis indicated that 3 dpi represents a critical window for secondary pathological responses, at which lactylation-related, ferroptosis-related, and inflammation-related signature scores were simultaneously elevated and showed the strongest inter-correlation. This finding is consistent with previous observations that the peri-3 dpi period is a highly active phase of immune responses and metabolic remodeling after SCI (Wu et al., 2013; Faden and Hoffman, 2006). Local ischemia, hypoxia, hemorrhage, and tissue destruction after SCI may collectively promote glycolytic remodeling, lactate accumulation, iron overload, lipid peroxidation, and inflammatory pathway activation (Lauro and Limatola, 2020; Zhang et al., 2019; Bie et al., 2025; Chen et al., 2023).

In the present study, KEGG and GSEA analyses enriched pathways such as ferroptosis, HIF-1 signaling, NOD-like receptor signaling, and cytokine-cytokine receptor interaction, providing pathway-level evidence that metabolic remodeling, ferroptosis-related stress, and inflammatory signaling are transcriptionally coordinated during the subacute phase after SCI. However, these analyses should be interpreted as evidence of co-activation and co-variation at the transcriptomic level rather than direct proof of causal molecular coupling among lactylation, ferroptosis, and neuroinflammation.

Following the tissue-level analyses, scRNA-seq localized the coordinated transcriptional signatures predominantly to pathological DAM-like microglial states. DAM lipid/metabolic and inflammatory DAM subpopulations expanded after SCI and were enriched for oxidative phosphorylation, lysosomal activity, glutathione metabolism, lipid processing, and ferroptosis-related pathways. The concept of disease-associated microglia was initially proposed in neurodegenerative disease contexts (Keren-Shaul et al., 2017), but recent studies indicate that DAM-like programs can also emerge rapidly after acute central nervous system injury (Milich et al., 2021; Masuda et al., 2019).

Pseudotime analysis further suggested an inferred transcriptional continuum from homeostatic-like microglia toward transitional and pathological DAM-like states. Along this inferred continuum, genes including Plin2, Lgals1, Lpl, Spp1, and Vim showed dynamic expression changes together with increasing lactylation-related, ferroptosis-related, and inflammation-related signature scores. These results support the interpretation that pathological DAM-like microglial states are closely associated with the coordinated transcriptional remodeling observed after SCI, rather than proving a discrete lineage transition or a single causal pathway.

Vim was prioritized as a candidate hub gene because it was identified by the bulk WGCNA-PPI pipeline, was associated with all three signature scores in pathological microglia, and remained prominent after single-cell expression stratification. VIM is a type III intermediate filament protein that has been implicated in cytoskeletal remodeling, migration, phagocytosis, inflammatory signaling, and lipid-associated stress responses in several cellular contexts (Jørgensen et al., 1993; dos Santos et al., 2015; Håversen et al., 2018; Jia et al., 2020; Gao et al., 2016). However, the current data do not establish that VIM directly regulates lactylation, ferroptosis, or inflammatory activation in SCI microglia.

Instead, the Vim-high transcriptional state was enriched for lysosomal, lipid-metabolic, phagocytic, stress-related, ferroptosis-related, and cytoskeletal-remodeling programs. The scTenifoldKnk analysis further provided an *in silico* prediction of candidate VIM-associated network perturbations rather than direct experimental evidence of VIM-dependent regulation. The predicted network included molecules such as Lgals3, Ftl1, Plin2, Fabp5, Pgk1, Timp1, and Hspb1, which are implicated in lipid metabolism, iron homeostasis, glycolysis, stress responses, and inflammatory activation. These findings support VIM as a candidate molecular node associated with pathological microglial remodeling and provide a rationale for direct functional studies.

The transcriptomic observations were supported by independent mouse tissue data and targeted *in vivo* validation. In the C5 contusion model, VIM, pan-Kla, ACSL4, and IL-1β were increased in whole-lesion spinal cord tissue at 3 dpi. Because Western blotting was performed using whole-lesion tissue lysates, these data reflect multicellular lesion-level responses and may include contributions from reactive astrocytes, infiltrating myeloid cells, fibroblast-like scar-associated cells, and other injury-reactive cell types. Therefore, the Western blot results should not be interpreted as microglia-specific protein changes.

Cell-type-associated spatial information was provided by immunofluorescence. VIM, pan-Kla, and ACSL4 signals showed increased spatial overlap within IBA1-positive cells after SCI. These findings are consistent with the transcriptomic association between VIM-related pathological myeloid-cell states and concurrent lactylation-related, ferroptosis-related, and inflammatory signatures, but they do not establish VIM-dependent regulation.

The human peripheral blood dataset GSE151371 was used only as an exploratory external reference for systemic SCI-associated immune expression patterns. Because this dataset was derived from circulating leukocytes rather than spinal cord tissue or cerebrospinal fluid, peripheral VIM expression cannot be considered a direct surrogate for CNS-resident microglial VIM. Peripheral VIM signals may reflect systemic immune activation, altered neutrophil or monocyte abundance, or generalized responses to acute trauma. Therefore, GSE151371 supports the systemic SCI relevance of candidate hub genes but does not establish CNS specificity or biomarker specificity for microglial VIM.

Several limitations should be acknowledged. First, the present study provides association-based and *in silico* evidence for a VIM-associated pathological microglial program, but it does not establish VIM-dependent causality. Although Vim was consistently prioritized by network analysis, transcriptional stratification, and *in silico* perturbation, microglia-specific Vim loss- and gain-of-function experiments will be required to determine whether VIM directly regulates lactylation-related transcriptional programs, ferroptosis susceptibility, or inflammatory activation after SCI.

Second, the interpretation of single-cell differential expression requires caution. Wilcoxon-based cell-level marker analyses were used for exploratory subcluster annotation and candidate-gene nomination, whereas module-score comparisons incorporated sample-aware mixed-effects models and sample-level summaries. Larger single-cell cohorts analyzed with formal pseudobulk or mixed-effects differential expression frameworks will be needed to further validate the cell-level DEG findings.

Third, the *in vivo* validation has inherent cell-type and molecular-resolution limitations. Whole-lesion Western blotting reflects multicellular tissue-level responses rather than microglia-specific protein changes, and pan-Kla immunoblotting does not identify specific lactylated proteins or modification sites. Similarly, ACSL4 upregulation and ferroptosis-related transcriptional signatures indicate ferroptosis-associated stress but do not independently establish ongoing ferroptotic cell death.

Finally, the human peripheral blood dataset GSE151371 cannot serve as a direct surrogate for VIM expression in CNS-resident microglia, and only male mice were used for *in vivo* validation. Future studies incorporating human SCI lesion-tissue or cerebrospinal-fluid datasets, female animals, additional injury stages, spatially resolved approaches, and direct Vim perturbation models will be important for defining the translational and mechanistic relevance of the VIM-associated program.

## 5 Conclusion

In conclusion, this study identifies coordinated lactylation-related, ferroptosis-related, and inflammation-related transcriptional signatures after SCI and localizes these signatures predominantly to pathological DAM-like microglial states. VIM was prioritized as a candidate hub associated with this pathological microglial program. These findings provide a candidate cellular and molecular framework for future mechanistic studies of secondary SCI. Direct functional experiments will be required to determine whether VIM is a causal regulator and a tractable therapeutic target.

### 5.1 Illustration of the multi-omics analytical workflow

The schematic overview depicts the integrative analysis pipeline combining bulk transcriptomics, single-cell RNA-seq, co-expression network analysis, and *in vivo* experimental validation.

## Data Availability

The original contributions presented in the study are publicly available. This data can be found in the Gene Expression Omnibus (GEO) repository with the accession numbers GSE47681, GSE5296, GSE162610, and GSE151371.
