# Supplementary material for: Multi-omics identifies a VIM-associated microglial program linked to lactylation, ferroptosis, and neuroinflammation after spinal cord injury
Source: Front Genet. 2026 Aug 27;17:1902559. doi: 10.3389/fgene.2026.1902559 (PMC13557701; doi:10.3389/fgene.2026.1902559)
Supplement: Supplementary file 1 [file Table3.docx]

**Supplementary Table 3. Statistical framework used for single-cell analyses**

Note. This table clarifies which analyses were sample-aware and which were used as exploratory cell-level screening. Cell-level Wilcoxon tests were used for marker nomination, subcluster annotation, and functional enrichment, and were not interpreted as standalone evidence for causal regulation. Module-score comparisons incorporated sample identity where applicable.

| **Analysis component** | **Main data / comparison** | **Statistical approach** | **Biological-replicate handling** | **Purpose in the study** | **Interpretation in the revised manuscript** |
| --- | --- | --- | --- | --- | --- |
| Global cell-type marker screening | All annotated cell types in GSE162610 | Seurat FindAllMarkers / FindMarkers; Wilcoxon rank-sum test; min.pct and logFC thresholds were used for marker screening. | Cell-level exploratory analysis; sample identity was retained in metadata but not used as a random effect for this marker-screening step. | To support cell-type annotation using canonical and data-driven marker genes. | Used for annotation and visualization only; not used as evidence for causal regulation or definitive biological-replicate-level differential expression. |
| Cell-type module-score comparison | Lactylation-related, ferroptosis-related, and neuroinflammation-related AddModuleScore values across cell types and time points. | Cell-level distributions were visualized; linear mixed-effects model: score ~ group + (1 \| sample); sample-level mean scores were also calculated. | Sample identity was modeled as a random effect; sample-level mean scores provided a biological-replicate-level summary. | To evaluate whether the three transcriptional signatures were enriched in specific cell types after SCI. | Interpreted as sample-aware evidence for coordinated signature enrichment, with cell-level plots used for visualization. |
| Microglial subcluster marker screening | Refined microglial subclusters after re-clustering of microglia. | Seurat FindAllMarkers / FindMarkers; Wilcoxon rank-sum test. | Cell-level exploratory analysis; biological sample structure acknowledged as a limitation for DEG inference. | To annotate microglial subclusters and define DAM lipid/metabolic and inflammatory DAM states. | Used as exploratory marker evidence for subcluster annotation and downstream enrichment, not as confirmatory pseudobulk DEG evidence. |
| Combined DAM vs. True Homeostatic MG I marker screening | Combined DAM = DAM lipid/metabolic + inflammatory DAM; compared with True Homeostatic MG I. | Seurat Wilcoxon rank-sum test with adjusted P-value and logFC thresholds. | Cell-level exploratory marker screening; no pseudobulk DESeq2/edgeR model was applied for this comparison. | To nominate genes and pathways associated with pathological DAM-like microglial states. | Interpreted cautiously as exploratory cell-level marker evidence; conclusions were supported by convergent bulk, network, and in vivo validation. |
| Pseudotime analysis | All microglia ordered using Monocle2, with True Homeostatic MG I as the root reference. | Monocle2 trajectory inference and pseudotime-associated gene analysis. | Trajectory inference was conducted at the cell level and interpreted as an inferred transcriptional continuum. | To describe state-continuum organization of microglia after SCI. | Interpreted as an inferred transcriptional state continuum rather than experimentally proven lineage conversion. |
| Vim-associated candidate-gene prioritization | Genes upregulated in combined DAM and correlated with optimized module scores; intersected with bulk WGCNA-PPI hub genes. | Spearman correlation with lactylation-related, ferroptosis-related, and neuroinflammation-related scores; intersection with bulk WGCNA-PPI hub list. | Correlation was cell-level and exploratory; candidate prioritization relied on convergence across independent analytical layers. | To prioritize a candidate hub gene associated with the three pathological signatures. | Vim was interpreted as a candidate hub associated with the signatures, not as a proven causal regulator. |
| Vim-low / Vim-mid / Vim-high stratification | Non-Sham combined DAM cells re-clustered and ranked by cluster-level median Vim expression. | Operational cluster-level stratification based on median Vim expression of re-clusters. | Cluster-level ranking was used to reduce the influence of single-cell dropout; it does not replace biological-replicate-level differential testing. | To identify VIM-associated pathological microglial transcriptional states within post-injury combined DAM cells. | Interpreted as operational state stratification for exploratory functional annotation. |
| Vim-high vs. Vim-low DEG analysis | Vim-high clusters compared with Vim-low clusters; Vim-mid retained for visualization but excluded from DEG comparison. | Seurat FindMarkers; Wilcoxon rank-sum test; GO, KEGG, and GSEA were performed on exploratory DEGs. | Cell-level exploratory comparison; formal pseudobulk or mixed-effects DEG modeling was not applied. | To annotate biological programs enriched in Vim-high pathological microglia. | Interpreted as exploratory functional annotation, not as definitive biological-replicate-level differential expression or causal evidence. |
| Vim expression-score correlation | Continuous Vim expression correlated with optimized lactylation-related, ferroptosis-related, and neuroinflammation-related scores within combined DAM cells. | Spearman correlation; residual-based partial-like correlation controlling for group and re-cluster where applicable. | Cell-level association analysis with adjustment for group and cluster structure in the residual-based analysis. | To assess whether Vim expression covaried with the three transcriptional signatures. | Interpreted as association only; correlation does not establish regulatory directionality. |
| In silico Vim perturbation | scTenifoldKnk analysis in Vim-high pathological microglia when sufficient cells were available; otherwise all non-Sham combined DAM cells were used. | scTenifoldKnk virtual perturbation; predicted perturbation-associated genes were intersected with exploratory Vim-high vs Vim-low DEGs. | Predictive single-cell network analysis; not an experimental loss-of-function assay. | To nominate candidate VIM-associated network components for future mechanistic testing. | Interpreted as hypothesis-generating in silico evidence; not equivalent to experimental Vim knockdown, knockout, or rescue validation. |

Abbreviations: DAM, disease-associated microglia; DEG, differentially expressed gene; GSEA, gene set enrichment analysis; LMM, linear mixed-effects model; PPI, protein-protein interaction; SCI, spinal cord injury; WGCNA, weighted gene co-expression network analysis.
